# Visible-light-assisted multimechanism design for one-step engineering tough hydrogels in seconds

**DOI:** 10.1038/s41467-020-18145-w

**Published:** 2020-10-05

**Authors:** Cong Wang, Ping Zhang, Wenqing Xiao, Jiaqi Zhao, Mengting Shi, Hongqiu Wei, Zhouhu Deng, Baolin Guo, Zijian Zheng, You Yu

**Affiliations:** 1 Key Laboratory of Synthetic and Natural Functional Molecule Chemistry of the Ministry of Education, College of Chemistry and Materials Science, Northwest University, 710069 Xi’an, China; 2 School of information science and technology, Northwest University, 710069 Xi’an, China; 3 Frontier Institute of Science and Technology, and State Key Laboratory for Mechanical Behavior of Materials, Xi’an Jiaotong University, 710049 Xi’an, China; 4 Nanotechnology Center, Institute of Textiles and Clothing, The Hong Kong Polytechnic University, Hung Hom, Kowloon, Hong Kong China

**Keywords:** Mechanical properties, Gels and hydrogels, Polymers

## Abstract

Tough hydrogels that are capable of efficient mechanical energy dissipation and withstanding large strains have potential applications in diverse areas. However, most reported fabrication strategies are performed in multiple steps with long-time UV irradiation or heating at high temperatures, limiting their biological and industrial applications. Hydrogels formed with a single pair of mechanisms are unstable in harsh conditions. Here we report a one-step, biocompatible, straightforward and general strategy to prepare tough soft hydrogels in a few tens of seconds under mild conditions. With a multimechanism design, the network structures remarkably improve the mechanical properties of hydrogels and maintain their high toughness in various environments. The broad compatibility of the proposed method with a spectrum of printing technologies makes it suitable for potential applications requiring high-resolution patterns/structures. This strategy opens horizons to inspire the design and application of high-performance hydrogels in fields of material chemistry, tissue engineering, and flexible electronics.

## Abstract (web summary)

Tough hydrogels capable of efficient mechanical energy dissipation are difficult to be patterned and/or printed which limits their application. Here the authors show a one-step, biocompatible and general strategy to prepare tough soft hydrogels with high-resolution 2D/3D microstructures quickly under mild conditions.

## Introduction

The engineering of tough hydrogels for widespread applications in diverse areas such as wearable electronics^1–4^, tissue engineering^5–7^, and protein/cell delivery^8–10^, has attracted intensive attention because of the unique mechanical properties of these materials. Typically, tough hydrogels have interpenetrating rigid and soft elastomeric networks that give them high mechanical strength and the ability to withstand strains to maintain integrity. When loading strains, rigid networks are rapidly disrupted to dissipate mechanical energy via different dissipation mechanisms^11–13^, which can effectively prevent catastrophic crack propagation in hydrogels. After releasing strains, deformed hydrogels can recover to original states with the assistance of their soft elastomeric networks. However, tough hydrogels are challenging to be fabricated as traditional hydrogels with single networks due to the contrasting multinetworks in one system.

In this field, such hydrogels with double networks, for example, are generally prepared by (i) diffusing a second monomer into a rigid network of the first polymer and polymerizing by heating or UV irradiation^14–17^, (ii) constructing secondary soft elastomeric networks in aqueous solutions of polymers, and then freezing them and/or soaking in ion solutions to form rigid primary networks^18–21^, or (iii) introducing microgels/fibres, jellyfish gel-like structures, and other energy dissipation mechanisms into second soft elastomeric networks^22,23^. Recently, one-pot synthesis and in situ formation of secondary networks are employed as efficient and straightforward strategies for preparing tough hydrogels. However, reaction times >1 h are still needed to complete the whole process. Long-time UV irradiation, ^60^Co-γ ray irradiation, or heating at high temperatures is extensively used to promote network formation but can hamper biological and industrial applications of tough hydrogels. Non-selective thermal-initiated and spontaneous polymerizations are inconvenient for printing complex 2D and 3D microstructures with high resolutions, which are needed for advanced applications in tissue engineering, ionotronics, etc. Moreover, a single pair of energy dissipation mechanisms that relying on single ionic-crosslinked, supramolecular or another rigid network to dissipate mechanical energy is sometimes ineffective in harsh environments due to the partial disruption of unstable rigid works in hydrogels, which considerably decreases their toughness^23,24^. The development of general and rapid methods for one-step fabrication of tough hydrogels with excellent biocompatibility, printability, and shape-flexibility is therefore important and presents a significant challenge.

Here, we report the design of a multimechanism, one-step, straightforward, and versatile method for developing tough hydrogels under mild conditions. Only a few tens of seconds are necessary to complete the whole fabrication process under visible-light irradiation. Rapid decomposition of the initiators makes it biocompatible and suitable for cell encapsulation and proliferation. In the proposed method, typical metal ions and commonly used synthetic polymers are generally used to form tough hydrogels with alginate. Such soft hydrogels have good mechanical strength and comparable toughness. The multimechanism design enables an increase in the hydrogel elasticity, a decrease in its plastic deformation, and retention of high toughness in various environments. Moreover, this light-assisted strategy is compatible with many different lithography techniques for manufacturing high-resolution 2D/3D microstructures (~100 µm). To the best of our knowledge, this is a rapid, one-step fabrication of tough hydrogels via a visible-light-assisted multimechanism design (Supplementary Table 1).

## Results

### One-step fabrication of tough, soft hydrogels with a multimechanism design

Tough hydrogels have been used in a wide range of applications in the last 20 years because of their excellent mechanical properties. A large number of previous studies have shown that covalent and ionic crosslinking reactions are simple and versatile routes for hydrogel network construction. However, such hydrogels with multiple contrasting networks are difficult to be fabricated, patterned, and printed by the methods used for traditional hydrogels with a single network (Supplementary Table 1), and this limits their specific applications in fields such as bioelectronics, energy, and tissue engineering. In terms of material design, the great challenges in this area are as follows. (i) The simultaneous construction of different networks via covalent and ionic crosslinking reactions in one pot must be achieved. (ii) The crosslinking reactions must be sufficiently fast and controllable for scalable fabrication, patterning, and printing of tough hydrogels. (iii) All the starting materials must be easily synthesized in a single step or preferably commercially available. (iv) The strategy must be general, straightforward, and biocompatible for applications such as cell encapsulation and proliferation. (v) As-prepared tough hydrogels should have excellent mechanical stability and toughness in various environments, e.g., different pHs and metal ions.

To meet these requirements, we have developed a strategy for one-step fabrication of tough hydrogels with the visible-light-assisted multimechanism design (THVMD) in seconds. This advanced fabrication strategy includes three orthogonal photoreactions, namely phenol-phenol coupling^25^, traditional radical polymerization, and ionic crosslinking for respectively constructing three different crosslinked networks^26^. As shown in Supplementary Fig. 1, the precursor solutions consisted of tris(2,2′-bipyridyl)dichlororuthenium(II) (Ru(II))/ammonium persulfate [Ru(II)/S_2_O_8_^2−^), ethylenediaminetetraacetic acid-chelated metal ions (EDTA-M), phenol-containing polymers (silk fibroin, gelatin or bovine serum albumin), alginate and acrylamide, which served as a visible-light initiator, metal ion source, and hydrogel precursors, respectively. Phenol-containing polymers and guluronic acid-containing alginate are respectively used to construct rigid Ph–N and metal–ion-crosslinked networks (M–N). Various monomers were used for creating soft elastomeric polymer networks (P–N). In these hybrid hydrogels, a densely physically crosslinked M–N can dissipate mechanical energy efficiently, and sparsely chemically crosslinked Ph–N and P–N enable a sample to return to its original configuration after deformation. The results in Supplementary Fig. 2 show that we successfully fabricated three tough, soft hydrogels.

**Fig. 1:**
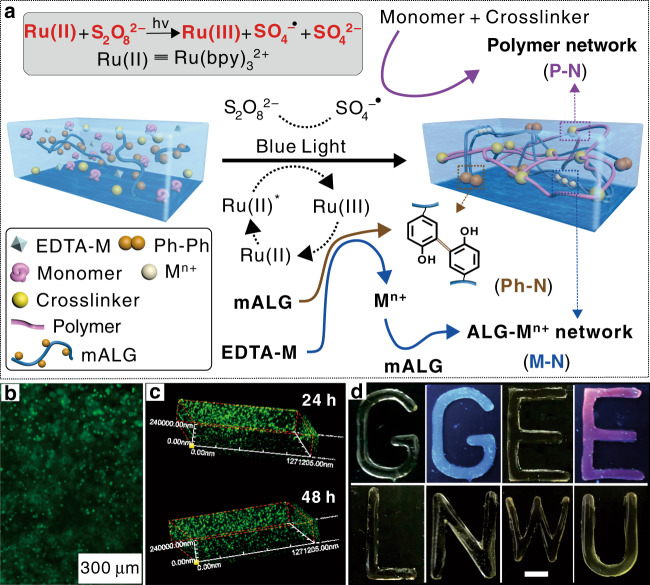
One-step fabrication of THVMD hydrogels. **a** The proposed schematic illustration for preparing tough hydrogels. **b** Fluorescent image of propidium iodide and fluorescein diacetate co-staining cells in the solution after irradiation. **c** Confocal fluorescent images of cells after 24 and 48 h incubation in hydrogels. **d** THVMD hydrogels under daylight and UV light irradiation. Letters of “G, E and L”: Hydrogels with different M–N networks of Tb^3+^, Eu^3+^, and Al^3+^, respectively; Letters of “N, W and U”: Hydrogels with different P–N networks of PEG, PAAm and PNIPAM, respectively. The scale bar is 1 cm.

To give the hydrogels high toughness and stability under harsh conditions, multimechanism materials were designed and prepared by using difunctional phenol-modified alginate (mALG) instead of pure alginate in the hydrogel precursors. When the unstable M–N is destroyed, the covalent double networks of Ph–N and P–N are retained and are still available for dissipating energy and maintaining the hydrogel integrity^22^. Based on this consideration and the achieved results, typical systems (Fig. 1), which contained Ru(II)/S_2_O_8_^2−^, EDTA-M, various monomers, and mALG were used to study the generality of this strategy methodically, and the hydrogel formation and toughening mechanisms involved. The patterning, 3D printing, and applications of the as-prepared tough hydrogels are discussed.

In terms of chemistry, the Ru(II) in the precursor solution is excited and S_2_O_8_^2−^ is simultaneously decomposed to sulfate anions and radicals on exposure to visible-light irradiation (~452 nm) (Fig. 1 and Supplementary Fig. 1)^25^. The generated radicals can initiate monomer polymerization. The excited Ru(II) is oxidized to Ru(III), which triggers the coupling of phenol groups and the release of M^*n*+^ from EDTA-M^26,27^. The free M^*n*+^ then rapidly forms ionic bonds with the guluronic acid units in mALG. A series of control experiments showed that all the components in the precursor solution and light irradiation are necessary for preparing such hydrogels (Supplementary Fig. 3).

A benefit of the fast decomposition of S_2_O_8_^2−^ under visible-light irradiation is that this method is non-toxic and biocompatible, and can be used for cell encapsulation and proliferation (Fig. 1b, c). Fibroblast cells (L929) were mixed with the fresh hydrogel precursor solution and immediately exposed to visible light. We did not observe dead cells during gelation and even observed a 260% increase in cell growth after incubation for 48 h (Supplementary Fig. 4). However, only around 86% of the encapsulated cells were alive after incubation for the same time in a similar system, in which UV irradiation was used (~10 min) to trigger the gelation process^28^. Importantly, this design principle can be readily used with typical M^*n*+^ ions, including Ca^2+^, Al^3+^ and luminescent lanthanide ions (i.e., Eu^3+^ and Tb^3+^), and commonly used synthetic polymers, including PEG, PAAm, and PNIPAM, to form transparent hydrogels with mALG (Fig. 1d). Consequently, the time for loading M^*n*+^ into the hydrogels greatly decreases from several hours to tens of seconds. To further understand the mechanism of this concept, THVMD hydrogels containing networks of polyacrylamide (P–N), Ca-crosslinked alginate (M–N), and coupled phenols (Ph–N) were selected as the model hydrogel for further study.

### Mechanism of hydrogel formation

Real-time in situ rheology was used to monitor the visible-light-triggered sol-gel transitions of the hydrogels. The gelation times (*t*_gel_) of the single network were 8, 40 and 190 s, respectively, which indicates that the order of the rates of network formation was P–N < M–N < Ph–N (Supplementary Fig. 6). The crosslinking densities were 0.7 mM (P–N) < 1.4 mM (Ph–N) < 28 mM (M–N); these were roughly calculated from the amounts of crosslinker added to the precursor solutions. We designed a model system to detect possible additional photoreactions during the gelation process (Supplementary Fig. 7). The low content of phenol residues in the PAAm-containing polymer showed that three networks, i.e., P–N, M–N and Ph–N, were independently formed even under the same light irradiation in one pot. These results enabled us to propose a reasonable mechanism for hydrogel formation (Supplementary Fig. 8). On light irradiation, Ph–N and M–N can sequentially and rapidly form in mALG. At the same time, P–N is built and interpenetrates with the double-crosslinked mALG hydrogel, which leads to the formation of THVMD hydrogels with a hierarchical network structure.

CaSO_4_ rather than CaCl_2_ is typically used as a Ca^2+^ source for constructing M–N, but a long waiting time (~2 h) is required to stabilize ionic crosslinking in the hydrogels^29^.

**Fig. 2:**
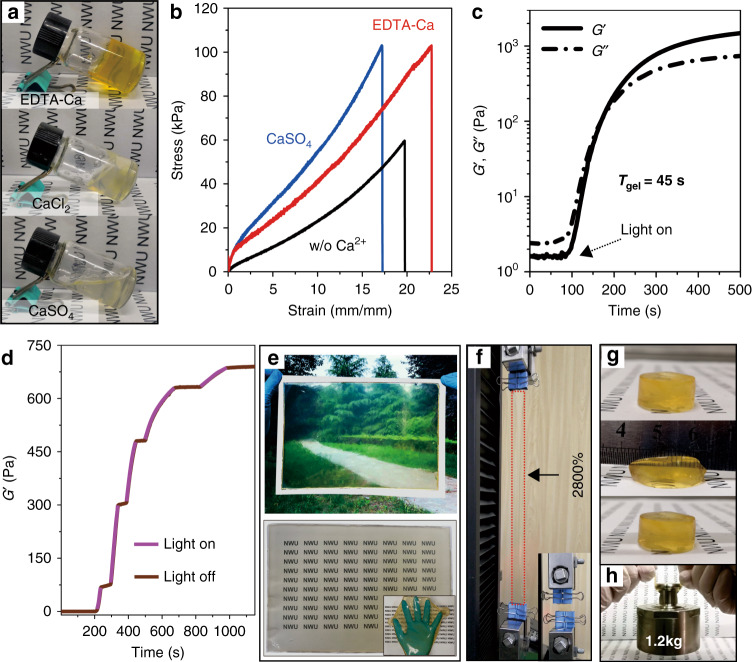
The formation mechanism of THVMD hydrogels. **a** Pure alginate hydrogels with different Ca^2+^ sources. [ALG] in all samples is 2.25 wt%. [EDTA-Ca] = 50 mM, [S_2_O_8_^2−^] = 131 mM, [Ru(II)] = 31.2 µM, 60 s irradiation at the intensity of 15 mW cm^−2^; [CaCl_2_] = [CaSO_4_] = 50 mM, 60 s waiting time. **b** Stress-strain curves of the PAAm hydrogel and tough hydrogels without Ph–N networks. [AAm] = 2.54 M, [MBA] = 0.7 mM. Concentrations of the other components used in these cases are the same as that in **a**. **c** Real-time in situ rheology of the hydrogel precursor upon continuous light irradiation. **d** Storage modulus (G′) measured with intermittent light irradiation. **e** The THVMD hydrogel film with a size of 13.5 inches on A4 PET and paper sheets. The inset shows the soft film peeled from the PET substrate. **f**, **g** Stretched, compressed hydrogels, and its ribbon bearing a weight of 1.2 kg, respectively.

In this study, EDTA-Ca, which is a better Ca^2+^ source than CaCl_2_ and CaSO_4_, was homogeneously dispersed in precursor solutions and rapidly decomposed with light irradiation, as mentioned above (Fig. 2a). It should be noted that the distribution of free Ca^2+^ ions at the molecular level enables the uniform formation of M–N in a short time and a noticeable increase in the stretchability of THVMD hydrogels (Fig. 2b). We found that the *t*_gel_ of these hydrogels was tunable, from 15 to 112 s, by varying the preparation conditions (Fig. 2c and Supplementary Fig. 9). A stepwise increase in the storage modulus (*G*′) was observed under intermittent light irradiation (Fig. 2d). This rapid and controllable gelation process can be ascribed to the following factors. (1) The three light-triggered orthogonal reactions can be controlled spatially and temporally. (2) Ru(II)/S_2_O_8_^2−^ is more efficient than Ru(II)/amines, Fe^2+^, and heating for initiating polymerization (Supplementary Fig. 10)^30–32^. (3) High-yield Ru(III)-triggered coupling of phenols is achieved in seconds^25^. (4) The rate of Ca^2+^ release is >20 times faster than those for riboflavin and enzyme catalysts, UV irradiation, and diffusion from a CaSO_4_ slurry (Supplementary Fig. 11)^19,33–35^. These advantages facilitate the rapid, large-scale fabrication of hydrogels, which is highly desirable for industrial applications. As a proof-of-concept, a precursor solution (~150 mL) was poured onto an A4 poly(ethylene terephthalate) sheet and then exposed to light irradiation for ~100 s. A uniform, transparent hydrogel film (13.5 inches) was prepared and was easily peeled from the original substrate (>85% at 550 nm, Fig. 2e). We also found that rapid gelation facilitated particle dispersion in the hydrogels, which effectively prevents particle settling in the slow thermal-induced gelation process (Supplementary Fig. 12). As expected, THVMD hydrogels have excellent flexibility, stretchability, compressibility, and can bear a load of ~4,000 times their own weight without failure (Fig. 2f–h, Supplementary Figs. 13 and 14).

### Mechanical properties of THVMD hydrogels

**Fig. 3:**
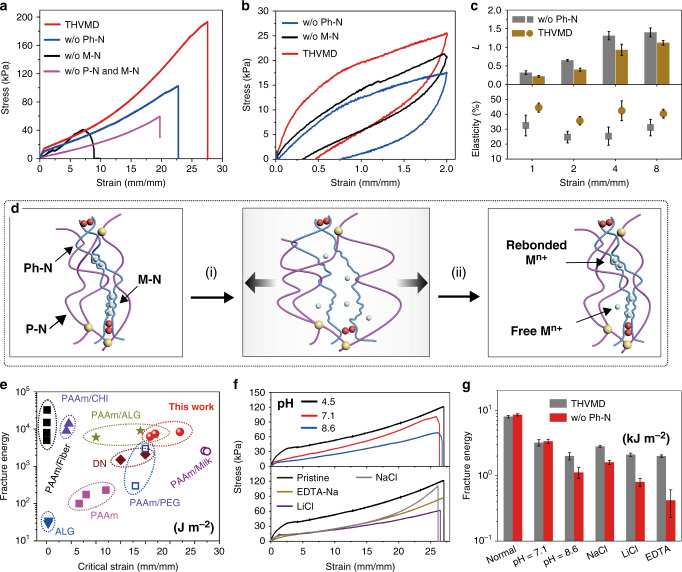
Mechanical properties of THVMD hydrogels under various conditions. **a** Stress-strain curves of various hydrogels. **b** Three types of hydrogels were stretched to a strain of 2 and were then released. **c** The plastic deformation (L) and elasticity of tough hydrogels and hydrogels without Ph–N networks at different strains. Error bars are defined as S.D. (*n* = 3 independent samples). **d** The proposed mechanism of (i) stretching and (ii) releasing-induced debonding/rebonding of M–N in THVMD. **e** Visual comparison of the fracture energy and maximum strain of tough hydrogels produced with different methods. **f** Stress-strain curves and **g** fracture energies of THVMD hydrogels and the hydrogels without Ph–N networks under different conditions. The concentrations of NaCl, LiCl and EDTA were 0.35, 0.47 and 0.1 M, respectively. Error bars are defined as S.D. (*n* = 3 independent samples). Notably, the water contents of all samples were kept at 80 wt% to reduce the effect of swelling on the mechanical testing.

The introduction of M–N and Ph–N significantly improved the stretchability and strength of THVMD. The proportions of the components in the precursor solutions had pronounced effects on the hydrogel mechanical properties (Supplementary Fig. 15). Under the optimum preparation conditions, the critical strain at rupture reached a maximum of 28 with the stress of ~200 kPa. These achieved values are, respectively, 1.4 and 3.4 times those of the hydrogel with the single network of P–N (Fig. 3a). Similar to other tough hydrogels, THVMD hydrogels can some extent recover to its original state after stretching/releasing because of partial reformation of M–N. After waiting for 30 min at room temperature, the work on reloading recovered to 60% of that for the first loading with a strain of 2, which is similar to that (68%) for the sample without Ph–N networks (Supplementary Fig. 16). We also found that the fracture energies of these hydrogels were as high as ~8000 J m^−2^, which is close to that reported for the tough hydrogels without Ph–N networks^19^ (Supplementary Fig. 17).

These network structures notably improved the elasticity and reduced plastic deformation of the hydrogels during the cyclic stretching/releasing process (Fig. 3b, c and Supplementary Fig. 18a, b). Rupture of M–N efficiently dissipated mechanical energy during the stretching process, and Ph–N and P–N enabled the hydrogels to rapidly return to their original shapes after release (Fig. 3d). The plastic deformation (*L*, Supplementary Fig. 19) of the hydrogels decreased by 20–40%, and their elasticity increased from ~25 to 45%, compared with those of hydrogels without Ph–N networks (Fig. 3c). When the strain increased (e.g., to 8), the Ph-Ns were fully stretched and then partially broken down, and the difference between the plastic deformations of the two types of hydrogel decreased. Nevertheless, the produced hydrogels maintained excellent elastic stability during repeated stretching/releasing cycles at this strain (Supplementary Fig. 18c–f). These results show that introducing a sparsely chemically crosslinked Ph–N into a hydrogel can efficiently enhance its elasticity and decrease its plastic deformation, with no apparent effect on the toughness. THVMD hydrogels therefore showed excellent stretchability with high fracture energies compared with the other hydrogels listed in Fig. 3e^36–40^.

More importantly, we found that hierarchical networks could provide hydrogels with high toughness in various environments. Considering the obvious effect of swelling behavior on the toughness of hydrogels in water (Supplementary Fig. 20), the content of water in THVMD was kept at 80 wt.% to accurately evaluate the changes of mechanical properties and toughness of hydrogels with varying pH and metal ions (see the toughness testing in the experimental section). Noticeable decreases in the stress were observed when the environmental pH and salts were varied, especially at low strains of 0–2.5, because the unstable ionic bonds of M–N were destroyed (Fig. 3f). However, THVMD still had fracture energies of 2000–3000 J m^−2^, i.e., 25%–45% of the values under normal conditions, because of the presence of covalently crosslinked Ph–N and Am-N. For example, EDTA is a well-known strong chelator for removing Ca^2+^ from hydrogels. When the hydrogel was exposed to an aqueous solution of EDTA (1:1 molar ratio of EDTA to EDTA-Ca), THVMD retained fracture energy of 2000 J m^−2^. Without Ph–N, this value sharply decreased by ~95% and the retained fracture energy was only several hundred joules per square metre, which is similar to that of a pure PAAm hydrogel (Fig. 3g)^19^. However, it is worth noting that the introduction of salts into the hydrogels caused a 2.0–3.5-fold increase in their ionic conductivities (Supplementary Fig. 21). Such excellent compressibility and conductivity enabled the fabrication of a capacitive pressure sensor with good responsivity (*S*_r_ ~ 4 kPa^−1^) and reliability under external pressures (Supplementary Fig. 22a, b). As well as a single sensor, a three-by-three sensor array can also be produced to record multiple pressure signals simultaneously, which is highly desirable for controlling electronic devices, as described below (Supplementary Fig. 22c, d).

### Printing 2D patterns and 3D structures

**Fig. 4:**
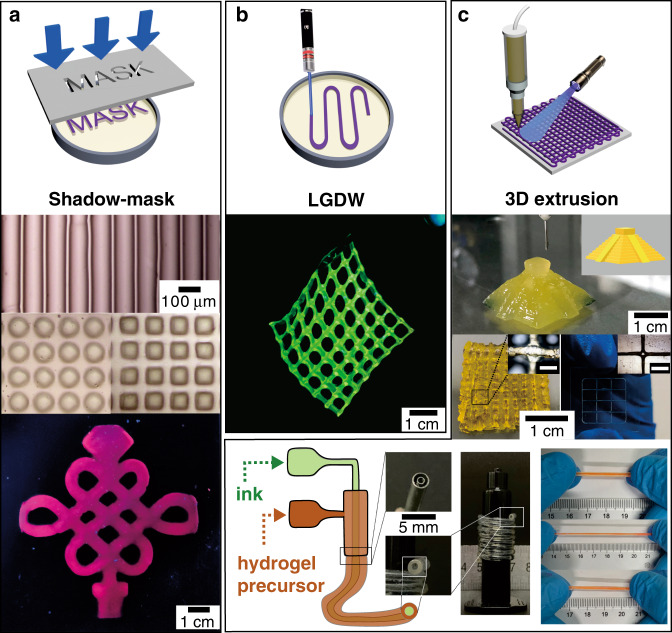
Designing THVMD patterns and 3D structures using typical printing techniques. **a** Shadow-mask lithography: Lines, balls, squares and Chinese knot. **b** Laser-guided direct printing: FITC-labelled mesh. The irradiation intensity = 200 mW cm^−2^, scanning rate = 90 mm s^−1^. **c** 3D extrusion printing: The pyramid-shape hydrogel, porous skeleton, grid (5 layers), and tough hydrogel tubes. The red solution in tubes is the ethylene glycol solution of Eosin Y. Both Eu-contained Chinese knots in **a** and FITC-labelled mesh in **b** fluoresce under UV irradiation (~365 nm). The thickness of samples in **a**, **b** is 2 mm. The scale bars in **c** are 1 mm.

The on-demand manufacture of tough hydrogels by the methods used for traditional hydrogels with a single network is still a challenging task. The key issues are the relatively slow gelation process and non-controllable hydrogel formation^22,23^. In the current study, we achieved the fabrication of THVMD hydrogels in seconds under visible-light irradiation. In addition to being suitable for shaping hydrogels in pre-designed moulds (Fig. 1d), this hydrogel preparation process is therefore readily compatible with a broad spectrum of printing techniques in terms of ink requirement, printing resolution, patterning strategy, and target applications. Figure 4 shows THVMD patterns, which were fabricated by various techniques, namely shadow-mask lithography, laser-guided direct writing (LGDW) lithography, and 3D extrusion printing. The patterns featured typical sub-100 µm resolution for shadow-mask photolithography and 3D extrusion printing, and sub-1 mm for LGDW with good shape-flexibility and were suitable for applications requiring high-resolution patterns/structures.

In the first two methods, hydrogels were selectively formed at the light-irradiated position (Fig. 4a, b). Lines, balls, squares, and Chinese knots were fabricated by shadow-mask lithography. The Chinese knots fluoresced brightly under UV-light irradiation because Eu^3+^–N was used as the sacrificial network in the hydrogel. The laser-assisted printing approach can be used to geometrically engineer hydrogels to give, for example, ribbons and mesh patterns (Fig. 4b and Supplementary Fig. 23a). The resolutions of these patterns can be tuned from millimetres for ribbons to several hundred micrometres for meshes by adjusting the laser point size. The deformation of a serpentine conductor during stretching was significantly reduced, compared to that of a straight conductor, which resulted in a smaller change in the resistance (Supplementary Fig. 23b). The abundant hydroxyl groups in mALG provided immobilizing sites for fluorescent molecules for labeling the printed scaffold (Fig. 4b). Because of its excellent mechanical properties, a FITC-labelled mesh was easily peeled from the substrate and handled by tweezers (Fig. 4b). We also produced pyramid-shaped and porous skeleton hydrogels by using 3D extrusion printing in combination with a visible-light-curing system. After extruding the solution from a nozzle, visible light immediately triggered THVMD formation and shaped the hydrogels in a layer-by-layer fashion (Fig. 4c and Supplementary Movie 1). The as-printed 3D hydrogel structure (e.g., a pyramid) had excellent compressibility and mechanical stability in 100 continuous compression/release cycles (Supplementary Fig. 24). Tough, stretchable hydrogel tubes can be fabricated by using a similar printing technique with a coaxial needle (18G/12G). However, this tubular structure of tough tubes was not obtained when printing them in a dark place due to the slow gelation process. With high biocompatibility, we can directly inject and encapsulate guest materials in tubes. These tough, soft hydrogel tubes are good candidates for artificial blood vessels for fabricating complicated organs in biomedical applications.

### Applications of THVMD hydrogels in ionotronics

**Fig. 5:**
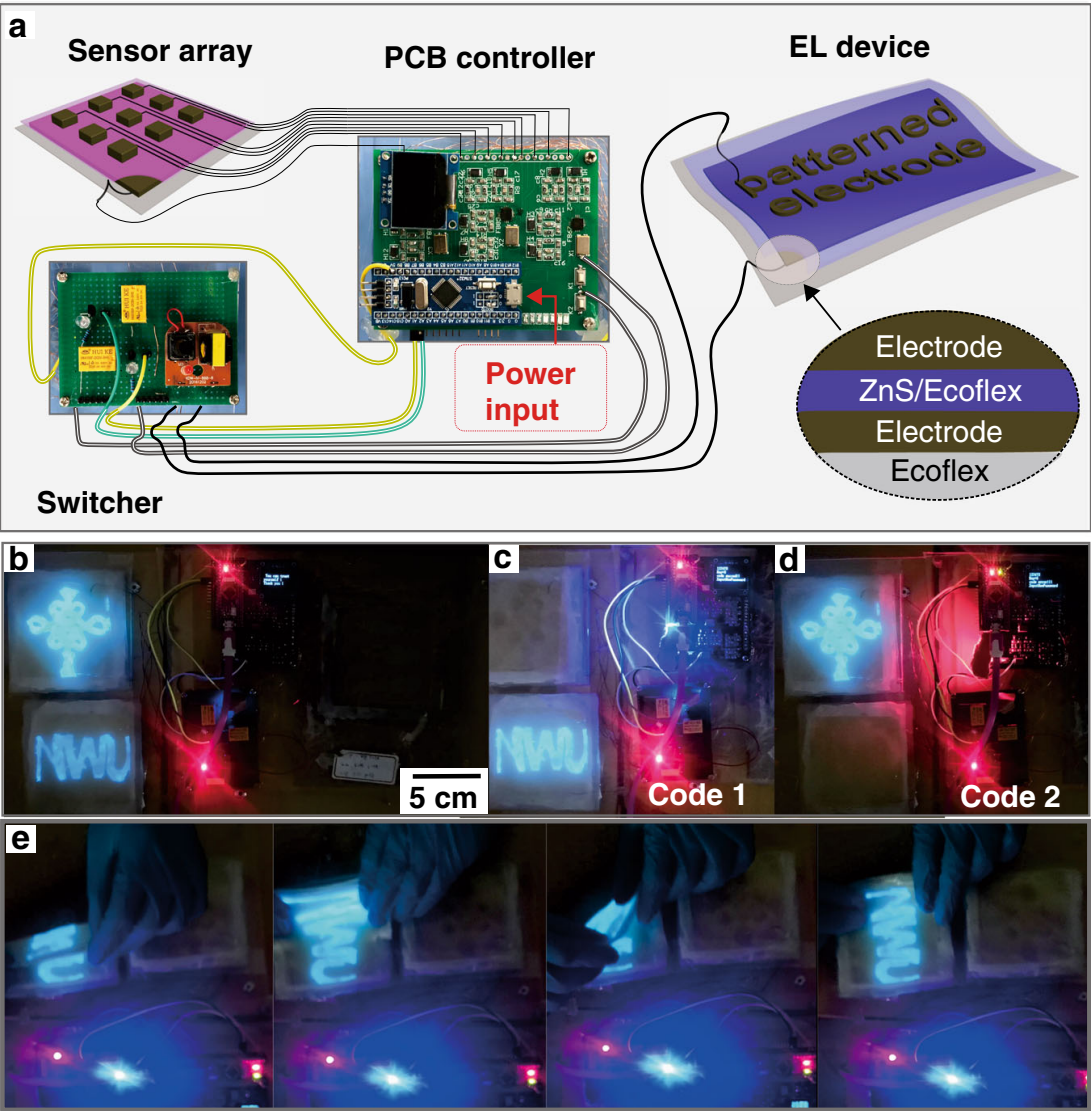
An integrated electronic system using THVMD hydrogel as transparent electrodes. **a** Schematic illustration of the fabrication of the fully stretchable electroluminescent device and the integration of all components into one system. **b**–**d** Operating and controlling the designed electronic system by inputting different codes. **e** The powered EL device under folding, stretching and twisting states.

The above discussions indicate that THVMD hydrogels show high toughness, stretchability, transparency, and conductivity, and have a broad compatibility with typical printing techniques. Unlike previously reported patterned hydrogel electrodes, which need an expensive laser cutter or other shaping equipment, patterned THVMD electrodes can be directly formed on the surfaces of devices via photolithography. These unique advantages inspired us to explore their potential applications in ionotronics to prolong the service life of electronics under large deformation. As a proof-of-concept, we designed an integrated electronic system that contained a switcher, a self-designed printed-circuit-board (PCB) controller, a THVMD -based sensor array, and electroluminescent (EL) units. The fully stretchable EL units were fabricated from 1 mm-thick hydrogel films for use as transparent electrodes, which allowed light to be transmitted (Fig. 5a). The top electrodes (i.e., a Chinese knot, and the letters of N, W and U) were prepared in advance by shadow-mask and LGDW techniques, respectively. The emitting layers (doped ZnS/Ecoflex, thickness ~100 µm) were then sandwiched between the bulk and patterned electrodes and sealed with Ecoflex films (~100 µm). Before assembly, thin PAAm layers of thickness several nanometres were coated on the Ecoflex and composite layers to achieve strong adhesion at the interfaces^41^. The application of an AC electric field caused blue luminescence within the semiconducting phosphor, corresponding to the dopants in the ZnS lattice (Fig. 5b). Because of the high conductivities of the hydrogels and thin emitting layer used, a working voltage as low as 110 V was enough for operating these EL devices. The states of the EL units can be controlled by inputting different codes. The hydrogel buttons on the sensor array were sequentially pressed (Supplementary Fig. 21c), and the capacitance changes were recorded and then translated to current signals by the PCB controller. The use of different input codes enabled control of the states of the EL units through the switcher (Fig. 5c, d and Supplementary Movie 2). Because tough, soft hydrogels were used in the system, the EL units can undergo deformation by folding, stretching, and rolling without any observed damage (Fig. 5e and Supplementary Movie 3).

## Discussion

In conclusion, we achieved the one-step preparation of next-generation, tough, soft hydrogels in seconds by using a rationally designed method that involved three orthogonal photoreactions in one polymeric system. THVMD hydrogels were prepared, patterned, and printed with high resolutions (~100 μm) by the same methods as traditional hydrogels with a single network. The designed multiple mechanism-method gives hydrogels with excellent mechanical properties and high toughness at harsh conditions. It can, therefore, be readily used for the production of flexible electronics such as capacitive sensors and EL devices, as shown in the current study.

To the best of our knowledge, this is an example of THVMD hydrogel production by this process (Supplementary Table 1). Typical issues in this field have been adequately addressed. Moreover, the proposed method includes, but not limited to, the following advantages in the preparation and applications of tough hydrogels. First, this design principle is generally applicable to synthetic polymers and typical M^n+^ ions. Smart, tough hydrogels can be prepared by introducing different environmental-responsive P–N and M–N networks^42^. With the multimechanism design, THVMD hydrogels have good mechanical properties even at harsh environmental conditions compared with the hydrogels with a single pair of networks. Moreover, the rapid Ru(II)-triggered release of M^*n*+^ from EDTA-M enables more efficient homogeneous ion dispersion in hydrogels than that achieved with the commonly used soaking method. Therefore, this controllable route for on-demand releasing M^n+^ via the degradation of EDTA-M can be extended as a typical method for distributing M^n+^ in materials, especially for the precursors that rapidly react with M^n+^.

Second, all starting materials are commercially available or can be easily synthesized in a single step. Therefore fabrication costs are reduced. For example, the price of the key component of Ru(II) is ~110 US dollars per gram, but only ~6 × 10^−5^ gram is used to fabricate one sample (40 × 15 × 1 mm) for a tensile test in this study. Phenol-contained materials are easily obtained from nature or prepared by the chemical post-modification with cheap phenol units (e.g, tyramine, tyrosine and hydroxyphenylacetic acid). Moreover, EDTA-M compounds are commercialized or synthesized by directly coordinating M^n+^ ions with ethylenediaminetetraacetic acid. On the other hand, tough hydrogels are one-step fabricated in seconds with reported multiple toughening mechanisms. The fabrication time is significantly decreased from >1 h to tens of seconds. Therefore, this strategy has broad potential applications in the field of material science from both materials and fabrication points of view.

Third, non-controllable and multistep fabrication processes make it challenging to pattern or print tough hydrogels with high resolutions. In this study, such light-controlled, rapid fabrication process is compatible with a broad range of lithography techniques. 2D and 3D microstructures with sub 100 µm are successfully made, which are vital components of electronic devices. More importantly, with its biocompatibility, this method can also be applied to encapsulate cells, proteins, and enzymes in shaped hydrogels for designing artificial tissues and organs. In view of all these advantages, we believe that this simple, general strategy and the prepared THVMD hydrogels will have a broad range of applications in fields such as material science, tissue engineering, optical devices, bioelectronics, and ionotronics.

## Methods

### Materials

Tris(2,2′-bipyridyl)dichlororuthenium(II) (Ru(II)) (Acros), sodium alginate (180947, G/M = 1.02, Sigma-Aldrich), poly(ethylene glycol) (~20,000 Da, Sigma-Aldrich), acrylamide (TCI), Ecoflex 00-30 (Smooth-On) and other chemicals (Sinopharm Chemical Reagent Co. Ltd.) were bought and used without further purification. EDTA-M (Eu^3+^, Tb^3+^ and Al^3+^) were synthesized by adding corresponding metal chloride or nitrate salts into aqueous solutions of ethylenediaminetetraacetic acid (EDTA) at the molar ratio of 1:1, refluxing for 12 h and then slowly cooling to room temperature to precipitate target products. Phenol-modified alginate (mALG), *N*-acryloyl glycinamide, poly(ethylene glycol) diacrylate (PEGDA) and silk fibroin solution were synthesized according to the reported methods, respectively^5,27,41,43^.

### Preparing hydrogels

To make the tough soft hydrogels, Ru(II), ammonium persulfate, EDTA-M, mALG, different monomers and *N*, *N*′-methylenebisacrylamide were firstly added to deionized water at predetermined concentrations. After degassing the solution in a vacuum chamber, the mixture was then carefully poured into a Teflon mould and cured under visible light (452 nm wavelength, 15 mW cm^−2^) for a predetermined time. 2D patterning hydrogels were prepared by placing shadow-masks on the top of the corresponding moulds before irradiation or fixing the laser pen onto a commercial 3D printer so that it can scan the surface (scanning rate: 90 mm s^−1^) and trigger the gelation at pre-designed orbits. After that, the unreacted places were gently rinsed by deionized water for several times to develop patterned hydrogels. To label the hydrogel, the as-printed mesh hydrogel was immersed into a 1 mg mL^−1^ dimethyl sulfoxide solution of fluorescein isothiocyanate (FITC) for 1 h at 40 ^o^C. Then, the excessed and physically absorbed FITC was removed by carefully rinsing with deionized water. As for 3D extrusion printing, a proper amount of Laponite XLG (5 wt%) was added in advance for thickening the mixture. After extruding from a 3D printer with a 21G, 31G or 18G/12G flat tip needle, the paste-like precursor alone or with the ink (55.5 wt% solution of poly(vinyl imidazolium) ethyl-sulfate salts in ethylene glycol) was immediately exposed to light irradiation (15 mW cm^−2^), forming 3D prototype hydrogels. In this study, the software of SolidWorks was adopted to design all orbits and models for laser-assisted and extrusion 3D printing.

### Cytocompatibility testing

mALG and PEGDA were dissolved in the Dulbecco’s Modified Eagle’s Medium (DMEM) with 10 % fetal bovine serum and sterilized in an oven at 55 ^o^C overnight. L929 fibroblast cells were suspended in the solution above and counted by a hemocytometer. Then the freshly aqueous solution of Ru(II)/S_2_O_8_^2−^ was quickly added and immediately exposed to an LED light (15 mW cm^−2^) for 5 min. After that, the cell-encapsulated hydrogels were incubated at 37 ^o^C with 5% CO_2_ for a determined time. The method of Alamar blue assay was used for quantitative analysis of the viability of cells. The dyed hydrogels were excited by a light beam at 560 nm, and the fluorescent intensities at 600 nm were recorded. The cell number in the transferred solution was calculated using the NIH ImageJ software after observing under inverted fluorescence microscopy (IX53, Olympus). Moreover, the morphology and distribution of cells in hydrogels were observed under a confocal laser microscope (FV1200, Olympus) and analysed using NIH ImageJ software.

### Rheology testing

All tests were performed on Anton Paar MCR302 at room temperature with a strain of 1% at 10 Hz. 1.6 mL of solution was firstly injected onto the plate (diameter of 50 mm) with a gap of 1,000 μm. Then, the rotor slowly went down and attached the solution surface. After that, visible light was applied through the transparent quartz plate (15 mW cm^−2^), and the storage and loss modulus were recorded immediately.

### Mechanical properties and toughness testing

All tensile tests were performed at room temperature with a speed of 100 mm min^−1^. Samples (40 × 15 × 1 mm) were adhered to the clampers using 3 M tapes. The compression tests were carried out at the speed of 10 mm min^−1^. The radius of the utilized cylindrical samples was 15 mm with a height of 10 mm. Furthermore, the tearing test was used to obtain fracture energy according to the previous literatures^8,19,44^. Hydrogels (20 mm × 2 mm × 50 mm) with a notch (30 mm) were fixed on the equipment and stretched at the speed of 100 mm min^−1^. A humidifier was used nearby to prevent the dehydration of samples.

To evaluate the effect of pH and salts on the mechanical properties, the as-prepared hydrogels were slowly dried at room temperature to keep the water content of hydrogels at 80 wt%. When the weight decreased by 50%, the same amount of corresponding solutions was added onto the hydrogel surfaces, following by a waiting period of 6 h to uniformly disperse solutions in hydrogels. After that, the samples were dried and swollen again according to the same procedure. Finally, the samples were tested in the same way as the fresh samples.

### Assembling the electronic system

For fabricating the capacitor array, a piece of THVMD hydrogel (2 wt% LiCl, 75 mm × 75 mm × 1.5 mm) was first placed onto a PET substrate. Then, nine small hydrogel tiles (15 mm × 15 mm × 1.5 mm) were fixed onto it and separated by a PET film with a thickness of 10 µm. These hydrogels were individually connected to the digital LCR meter (TH2819A) or the PCB unit by conductive metal wires. After that, the whole device was sealed by a PET film for preventing dehydration. As for the electroluminescent device, doped-ZnS (60 wt%) was mixed with Ecoflex 00-30 (A:B, 1:1), spin-coated on a PET sheet and dried at 80 ^o^C for 30 min. The ZnS/Ecoflex film (~100 µm) was then peeled off from the substrates and sandwiched between two THVMD hydrogel electrodes. The top electrodes were patterned by the shadow-mask (i.e., Chinese knot) and laser-assisted (i.e., NWU) printing techniques, respectively. Specially, thin PAAm hydrogel layers were constructed in advance on the surface of Ecoflex and its composites were used to improve the adhesion force between the layers^41^. The electrodes were individually connected to the switcher unit by metal wires. After that, the EL devices were sealed by thin Ecoflex (~100 µm) films to prevent the water evaporation from the hydrogel electrodes. Finally, all units were assembled to an electronic system that was controlled by inputting different codes.

Further details on the methods are available in the Supplementary Information.

## Supplementary information

Supplementary Information

Description of Additional Supplementary Files

Supplementary Movie 1

Supplementary Movie 2

Supplementary Movie 3

## Data Availability

The data that support the findings of this study are available from the corresponding author on request.
