# Supplementary material for: Visible-light-assisted multimechanism design for one-step engineering tough hydrogels in seconds
Source: Nat Commun. 2020 Oct 5;11:4694. doi: 10.1038/s41467-020-18145-w (PMC7536405; doi:10.1038/s41467-020-18145-w)
Supplement: Supplementary file 1 — Supplementary Information [file 41467_2020_18145_MOESM1_ESM.pdf]

Supplementary Information

Visible-light-assisted Multimechanism Design for One-step Engineering Tough Hydrogels in Seconds

Cong Wang[1, #], Ping Zhang[1, #], Wenqing Xiao[1, #], Jiaqi Zhao[2], Mengting Shi[3], Hongqiu Wei[1], Zhouhu Deng[2], Baolin Guo[3], Zijian Zheng[4] & You Yu[1,*]

Correspondence to: yuyou@nwu.edu.cn

Affiliations:

[1]Key Laboratory of Synthetic and Natural Functional Molecule Chemistry of the Ministry of Education, College of Chemistry and Materials Science, Northwest University, Xi'an 710069, China.

[2]School of information science and technology, Northwest University, Xi'an 710069, China.

[3]Frontier Institute of Science and Technology, and State Key Laboratory for Mechanical Behavior of Materials, Xi'an Jiaotong University, Xi'an, 710049, China.

[4] Nanotechnology Center, Institute of Textiles and Clothing, The Hong Kong Polytechnic University, Hung Hom, Kowloon, Hong Kong, China.

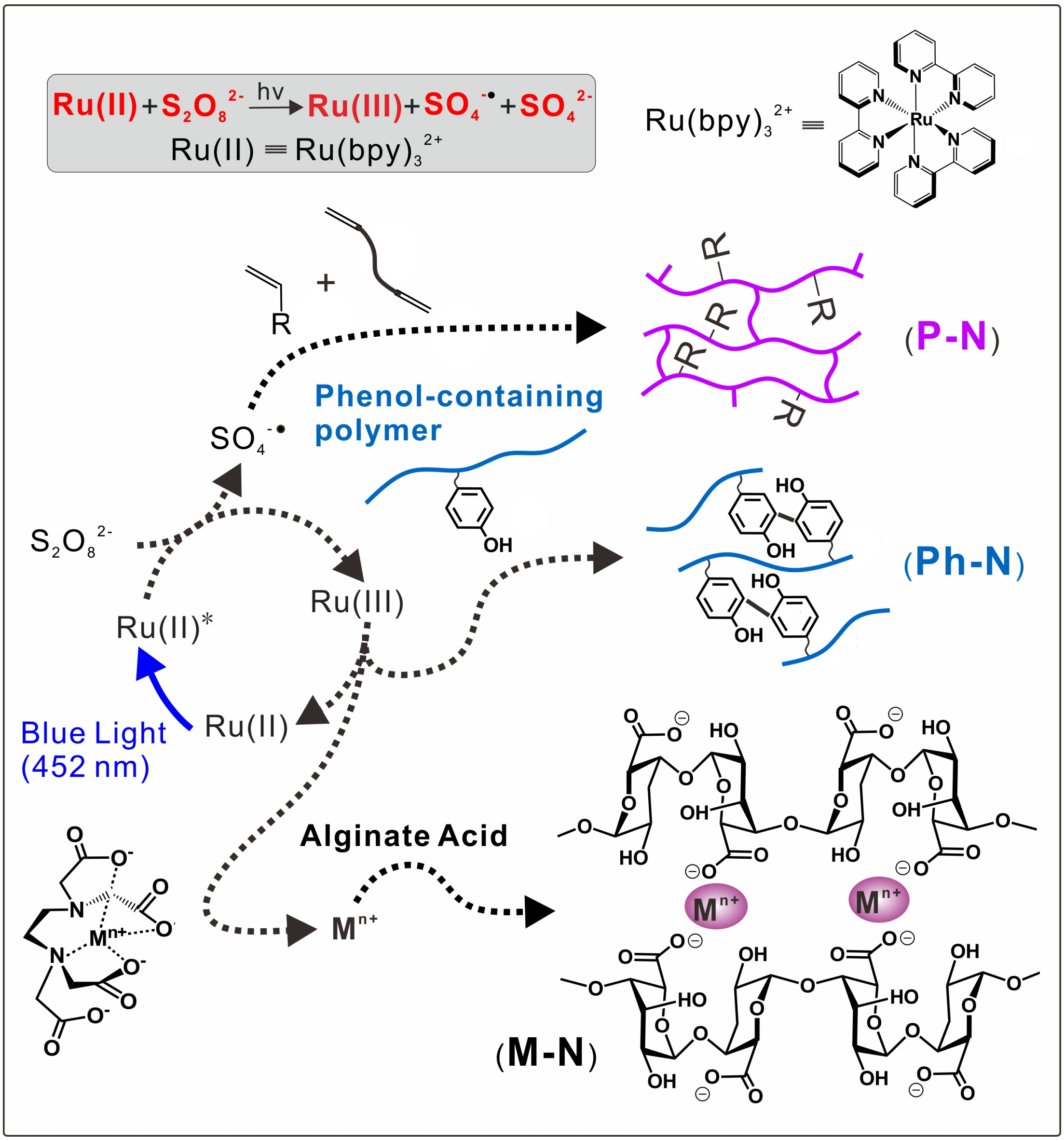

Supplementary Figure 1. The proposed three orthogonal photoreactions in THVMD hydrogels by the catalysis of Ru(II)/$S_2O_8^{2-}$.

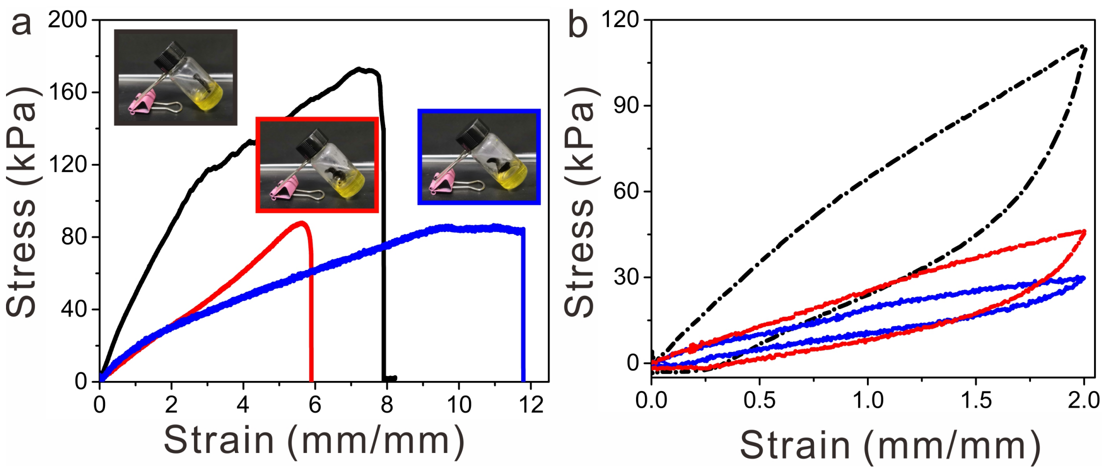

Supplementary Figure 2. One-step fabrication of THVMD hydrogels with multinetwork via three orthogonal reactions of radical polymerization, phenol coupling and ionic crosslinking. (**a**) Stress-strain curves of three tough hydrogels. (**b**) Hydrogels were each stretched to a strain of 2 and were then released. Black: gelatin (5 wt%); Red: Silk fibroin (7 wt%); Blue: Bovine serum albumin (5 wt%). Other components: [mALG] = 2.25 wt%, [EDTA-Ca] = 50 mM, [$S_2O_8^{2-}$] = 131 mM, [Ru(II)] = 31.2 µM, [AAm] = 2.54 M, [MBA] = 0.7 mM, 60 s irradiation at the intensity of 15 mW cm^{-2}.

Supplementary Note 1

To study the feasibility of this strategy for fabricating THVMD hydrogels via the proposed orthogonal photoreactions with the catalysis of Ru(II)/$S_2O_8^{2-}$. Three phenol-contained natural polymers, including gelatin, silk fibroin, and bovine serum albumin were used for constructing Ph-N networks of tough hydrogels via the phenol coupling reaction. AAm and MBA were used for building P-N networks via radical polymerization. Sodium alginate was used for creating M-N networks via the ionic-crosslinking reaction. Supplementary Figure 2a showed that these hydrogels were obtained in one step with short light irradiation, and presented good mechanical strength and stretchability. Moreover, the cyclic tensile tests in Supplementary Figure 2b indicated that there were obvious hysteresis during all stretching/release processes. The achieved results indicated that these THVMD hydrogels were tough, and can dissipate the mechanical energy when tensile strains were applied.

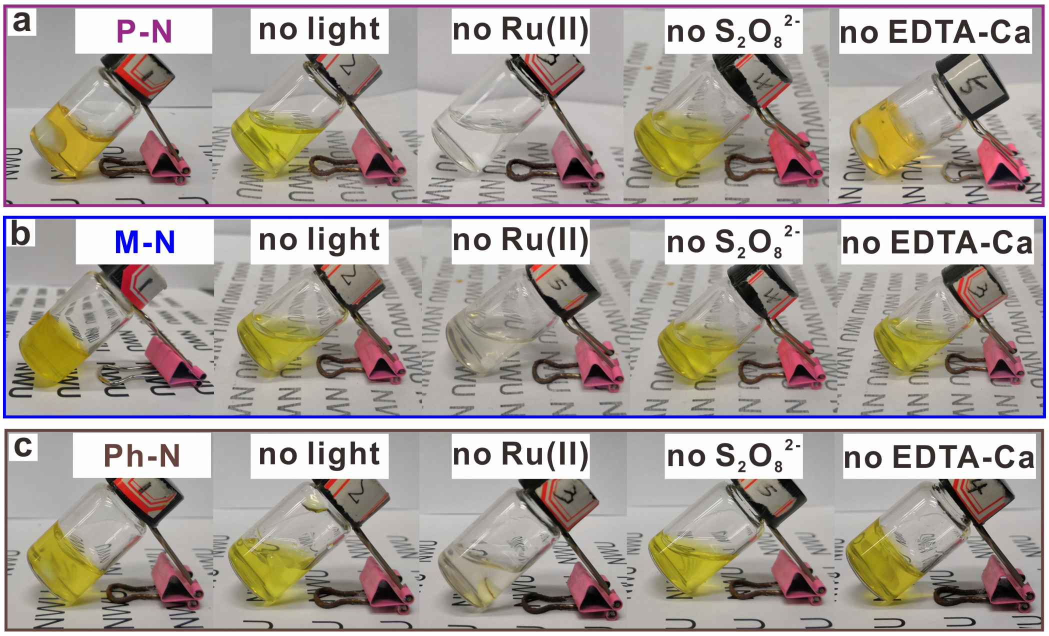

Supplementary Figure 3. Control experiments for preparing THVMD hydrogels. All hydrogels were prepared with a single network of P-N, M-N or Ph-N. Experiments were performed at room temperature under the irradiation intensity of 15 mW cm^{-2}. (**a**) For P-N, [AAm] = 2.54 M, [MBA] = 0.7 mM, [Ru(II)] = 31.2 μM and [$S_2O_8^{2-}$] = 131 mM; (**b**) For M-N, [ALG] = 2.25 wt%, [EDTA-Ca] = 100 mM, [Ru(II)] = 31.2 μM, [$S_2O_8^{2-}$] = 131 mM; (**c**) For Ph-N, [mALG] = 2.25 wt%, [Ru(II)] = 31.2 μM, [$S_2O_8^{2-}$] = 131 mM, respectively.

Supplementary Note 2

As shown in Supplementary Figure 3, a series of control experiments of the model system were performed to investigate the formation mechanism of THVMD hydrogels in this study. All hydrogels were prepared with a single network of P-N, M-N, or Ph-N. This design can clearly present the effect of fabrication conditions on the photoreactions of radical polymerization, phenol coupling, and ionic crosslinking reactions in hydrogels. Without the use of Ru(II), $S_2O_8^{2-}$ and light irradiation, it was found that all of the control samples were in liquid states, and no sol-gel transition was observed. With the absence of EDTA-M, the networks of P-N and Ph-N formed in Figures S3a and b. The reasons can be assigned for the use of MBA as the crosslinkers of P-N, and the coupled phenols in mALG that act as the crosslinking points of Ph-N. However, due to the lack of M^{n+} photo-released into precursor solutions, the M-N network didn’t form in Supplementary Figure 3c. These results indicated that typical monomers and EDTA-M, MBA, mALG, Ru(II)/$S_2O_8^{2-}$ and light irradiation were necessary to achieve the one-step fabrication of tough hydrogels with a multimechanism design.

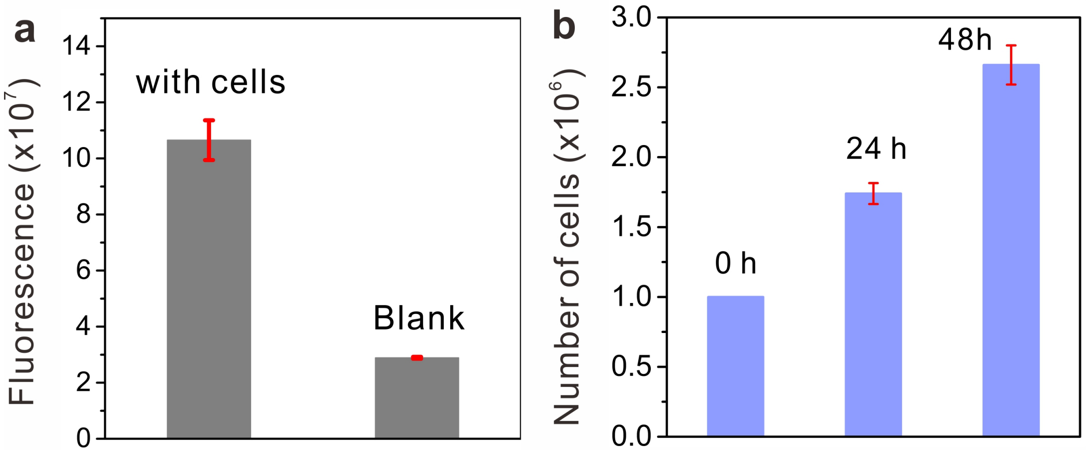

Supplementary Figure 4. Cytocompatibility test for the visible-light-assisted preparation of hydrogels. (**a**) The fluorescence of solutions before and after adding cells. (**b**) The number of cells encapsulated in hydrogels after 24 and 48 h incubation. Error bars are defined as S.D. (n = 3 independent samples).

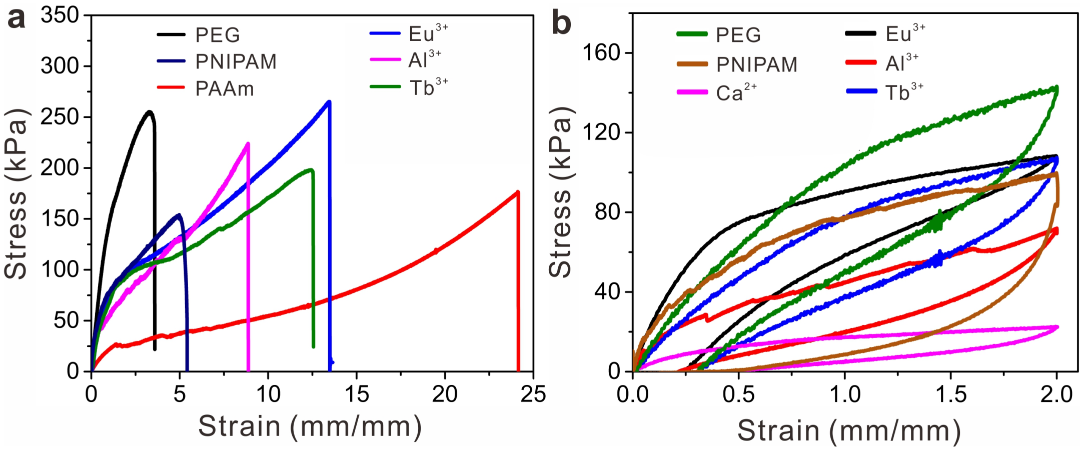

Supplementary Figure 5. Mechanical tests of different THVMD hydrogels. (**a**) Stress-strain curves of different THVMD hydrogels (**b**) One cycle of tensile tests of corresponding hydrogels in Figure **a**. All hydrogels were prepared at room temperature under the irradiation intensity of 15 mW cm^{-2}. Concentrations of the monomers of PEG, PNIPAM and PAAm were 2.54 M. [MBA] = 0.7 mM, [Ru(II)] = 31.2 µM, [$S_2O_8^{2-}$] = 131 mM, [EDTA-Ca] = [EDTA-Al] = [EDTA-Eu] = [EDTA-Tb] =100 mM, [mALG] = 2.25 wt%. When varying the monomers of synthetic polymers, the concentrations of [EDTA-Ca], mALG, [MBA], [Ru(II)] and [$S_2O_8^{2-}$] were kept constant. When varying metal ions, the concentrations of monomers, mALG, [MBA], [Ru(II)] and [$S_2O_8^{2-}$] were kept constant.

Supplementary Note 3

The stress-strain curves in Supplementary Figure 5a showed that different THVMD hydrogels were successfully prepared by introducing different monomers and metal ions to construct corresponding P-N and M-N networks. These hydrogels are stretchable and have good mechanical strength (150~250 kPa) with strains of 3~24. Moreover, the cyclic tensile tests showed that there was obvious hysteresis in all samples (Supplementary Figure 5b), which indicated the mechanical energy was efficiently dissipated via the disrupt of rigid networks in these hydrogels when releasing the strain from 2 to 0. On the basis of these achievements, we can conclude that this new strategy is general, straightforward, and is readily applicable for preparing THVMD hydrogels. The mechanical property and toughness are easily tuned by varying synthetic polymers and metal ions in hydrogels.

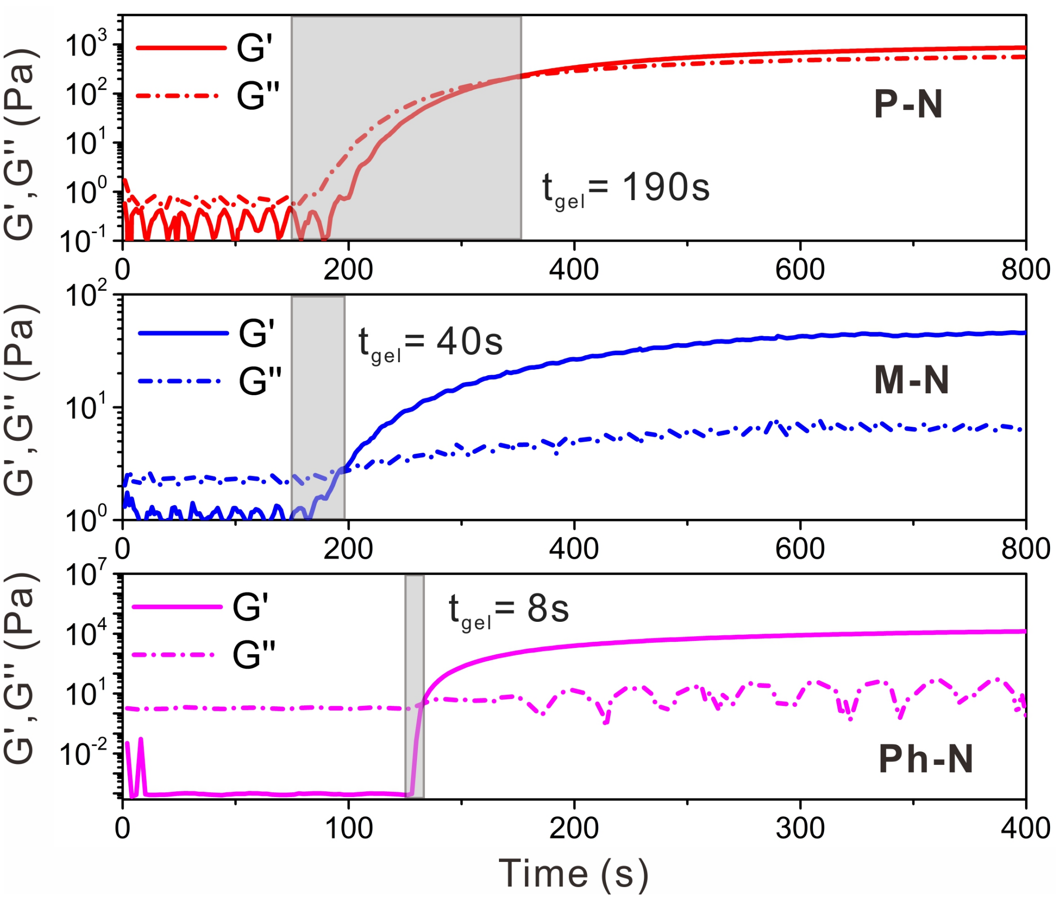

Supplementary Figure 6. Real-time in situ rheology tests of hydrogels with a single network of P-N, M-N or Ph-N. All experiments were performed at room temperature under the irradiation intensity of 15 mW cm^{-2}. For P-N, [AAm] = 2.54 M, [MBA] = 0.7 mM, [Ru(II)] = 31.2 μM and [$S_2O_8^{2-}$] = 131 mM; For M-N, [ALG] = 2.25 wt%, [EDTA-Ca] = 100 mM, [Ru(II)] = 31.2 μM, [$S_2O_8^{2-}$] = 131 mM; For Ph-N, [mALG] = 2.25 wt%, [Ru(II)] = 31.2 μM, [$S_2O_8^{2-}$] = 131 mM, respectively.

Supplementary Note 4

The rheology characterization was conducted to determine the gelation time of three hydrogels with single networks of P-N, M-N, and Ph-N under the same fabrication condition of THVMD hydrogels. 1.6 mL of the corresponding hydrogel precursor was firstly injected onto the plate with a gap of 1,000 μm. Then, the rotor slowly went down and attached the solution surface. After that, visible light was applied through the transparent quartz plate, and the storage (G′) and loss modulus (G″) were recorded immediately.

For each system, only one kind of crosslinking reaction proceeded that resulting in a single network in hydrogels. Briefly, in P-N, crosslinkers (MBA) were polymerized with monomers (AAm) and formed the covalently crosslinked PAAm hydrogels. In M-N, the photo-released Ca^{2+} from EDTA-Ca rapidly reacted with alginate and formed the ionic-crosslinked alginate hydrogels. It was worthy of note that the coupling reaction of phenol groups did not proceed due to the absence of phenols in pure alginate chains. Therefore, only the M-N network was formed at this reaction condition. In Ph-N, without the use of EDTA-Ca, the Ph-N network was created via the phenol-coupling reaction of mALG in hydrogels.

As shown in Supplementary Figure 6 (the shaded area), the gelation time was calculated from the irradiation starting to the crossover point of the G′ and G″ curves (G' = G″). The gelation times of these hydrogels were 190, 40 and 8 s for P-N, M-N, and Ph-N, respectively, at the same condition for preparing tough hydrogels. This result indicates that the gelation rates of hydrogels are P-N < M-N < Ph-N. Moreover, these aforementioned crosslinking reactions independently proceed in the precursor solution. Therefore, we can speculate that when preparing tough hydrogels at this condition, the network of Ph-N is first formed. Then, Ca^{2+} is released and crosslinked with alginate, forming an ionic crosslinked network. Finally, P-N is formed and interpenetrated into the Ph-N and M-N networks, resulting in tough hydrogels with multiple toughening mechanisms.

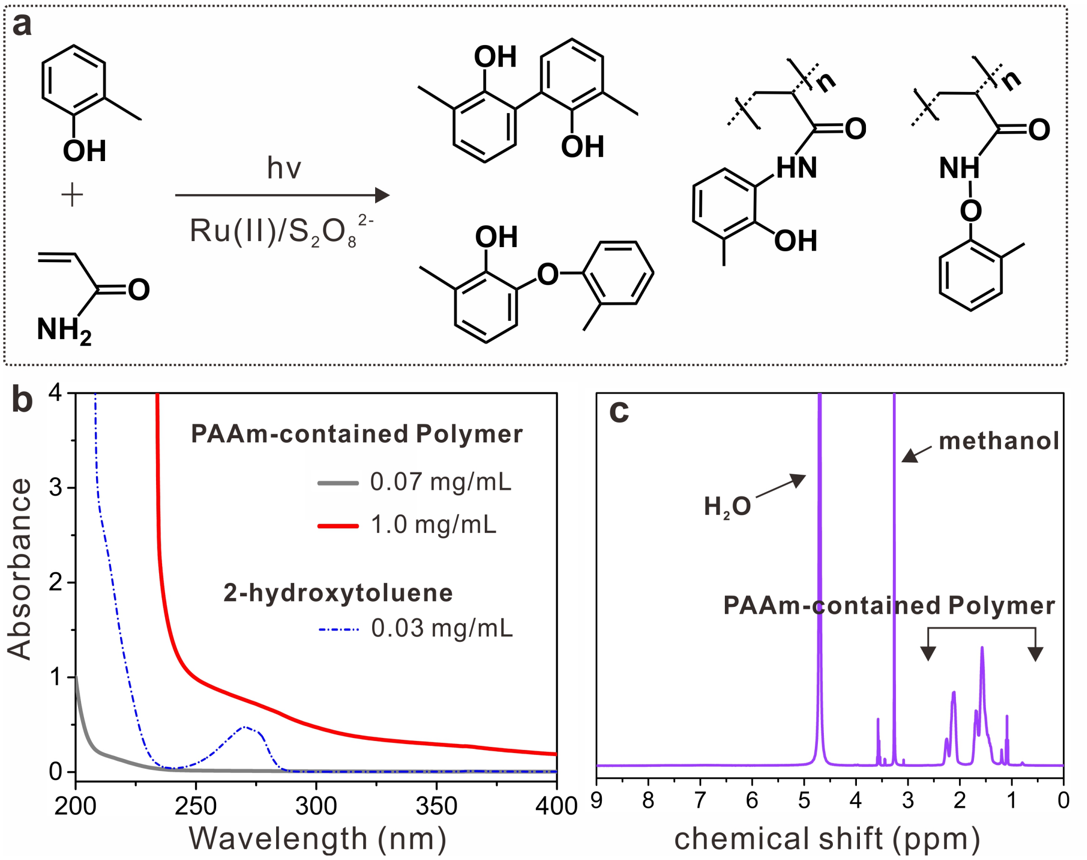

Supplementary Figure 7. The possible additional photoreaction during the gelation process. (**a**) Possible products generated between 2-hydroxytoluene and AAm. (**b**) UV-vis and (**c**) ^{1}H-NMR spectra of the PAAm-contained polymer (wt%). [2-hydroxytoluene] = 0.32 M, [AAm] = 2.54 M, [Ru(II)] = 31.2 μM, [$S_2O_8^{2-}$] = 65 mM, and the irradiation intensity was 15 mW cm^{-2}.

Supplementary Note 5

The 2-hydroxytoluene-contained control experiment was designed to detect the possible reaction between P-N and Ph-N. As shown in Supplementary Figure 7a, this phenol derivative supplies one reaction site to modify the polymer and themselves. The diphenol products are soluble in methanol that can be easily removed from the precipitation. Finally, only the PAAm-contained polymer was obtained. Therefore, the possible reaction between 2-hydroxytoluene and PAAm can be easily studied by using UV-vis and ^{1}H-NMR spectra. In detail, AAm, MBA, Ru(II), $S_2O_8^{2-}$, 2-hydroxytoluene was mixed in water with the pre-determined concentrations. After light irradiation for 100 s, the solution was first dropwise added into methanol to precipitate the PAAm-contained polymer. Then, the solid product was collected, re-dissolved into an aqueous solution of NaOH (1 wt%), and precipitated again in methanol. After repeating the same procedure three times, the product was dried in a vacuum. Figures S7b and c showed that there was no obvious characteristic absorption and peaks of phenol groups observed in the UV-vis and ^{1}H-NMR spectra. This result indicated that 2-hydroxytoluene did not react with AAm, and no phenol groups were grafted onto the PAAm chains. As for the hydrogels, the possible reaction between networks of P-N and Ph-N did not proceed via PAAm (in P-N) and phenols (in Ph-N). Therefore, we can speculate that three networks of P-N, M-N, and Ph-N independently formed even under the same light irradiation in one pot.

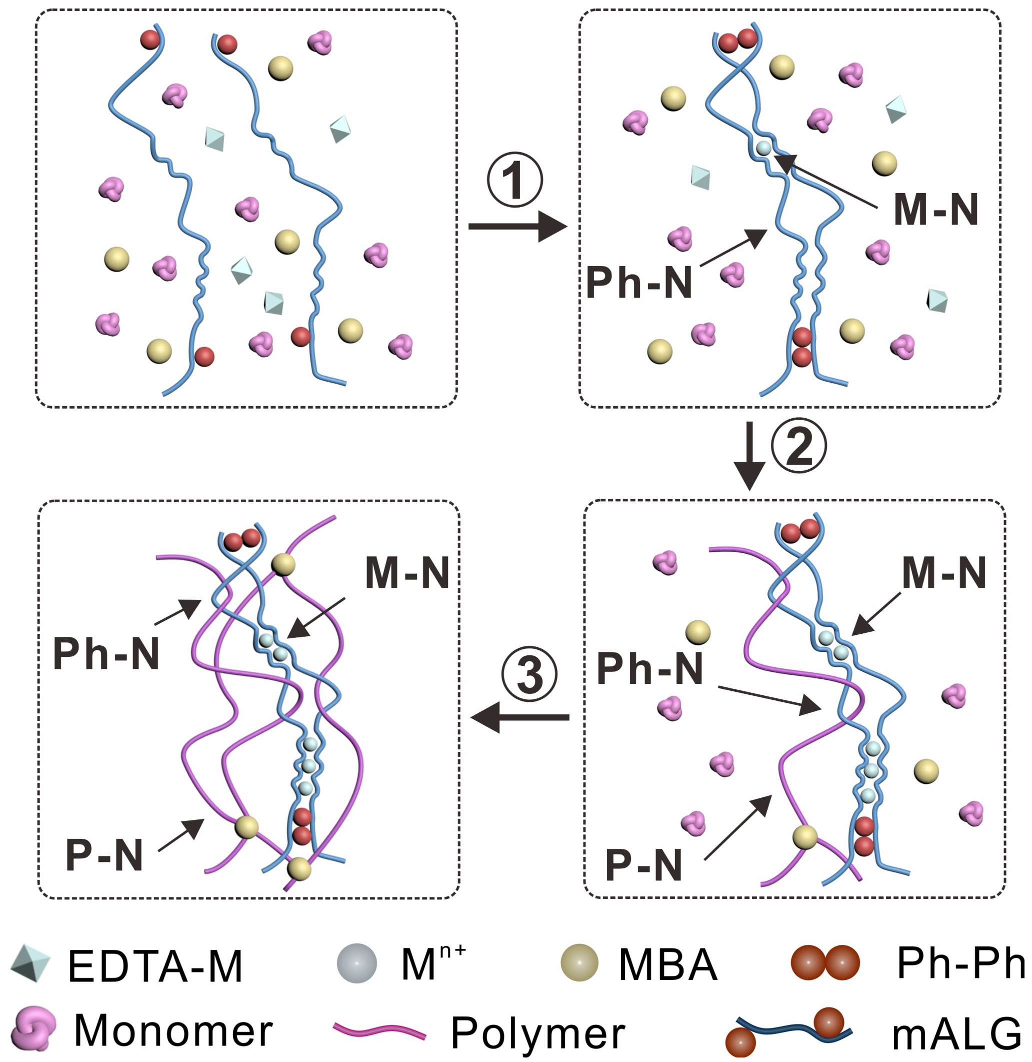

Supplementary Figure 8. The proposed mechanism for the formation of THVMD hydrogels under visible light irradiation. With light illumination, the coupling reaction of phenols in mALG proceed and the Ph-N was firstly formed in this system. Then, M^{n+} ions were photo-released from EDTA-M and triggered the formation of M-N in hydrogels. Last, the P-N network was formed via the radical polymerization of various monomers and interpenetrated into Ph-N and M-N networks. As a consequence, THVMD hydrogels were obtained with a multimechanism design.

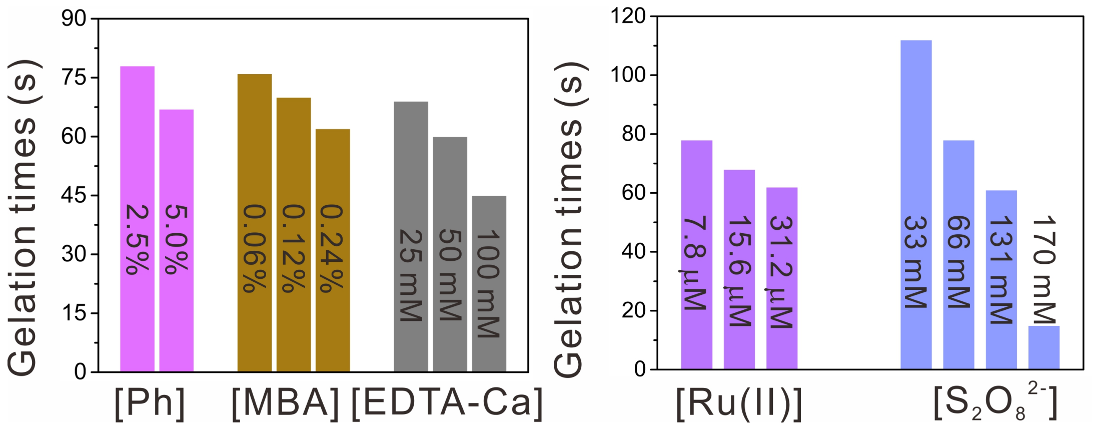

Supplementary Figure 9. Gelation times of hydrogel precursors at different conditions. All experiments were performed at the following conditions: [AAm] = 2.54 M, [MBA] = 0.7 mM, [EDTA-Ca] = 100 μM, [mALG] = 2.25 wt%, [Ru(II)] = 31.2 μM, [$S_2O_8^{2-}$] = 131 mM, the irradiation intensity = 15 mW cm^{-2}. For specifically evaluating the effect of preparation conditions on the gelation time, only one concentration was varied, and others were kept constant.

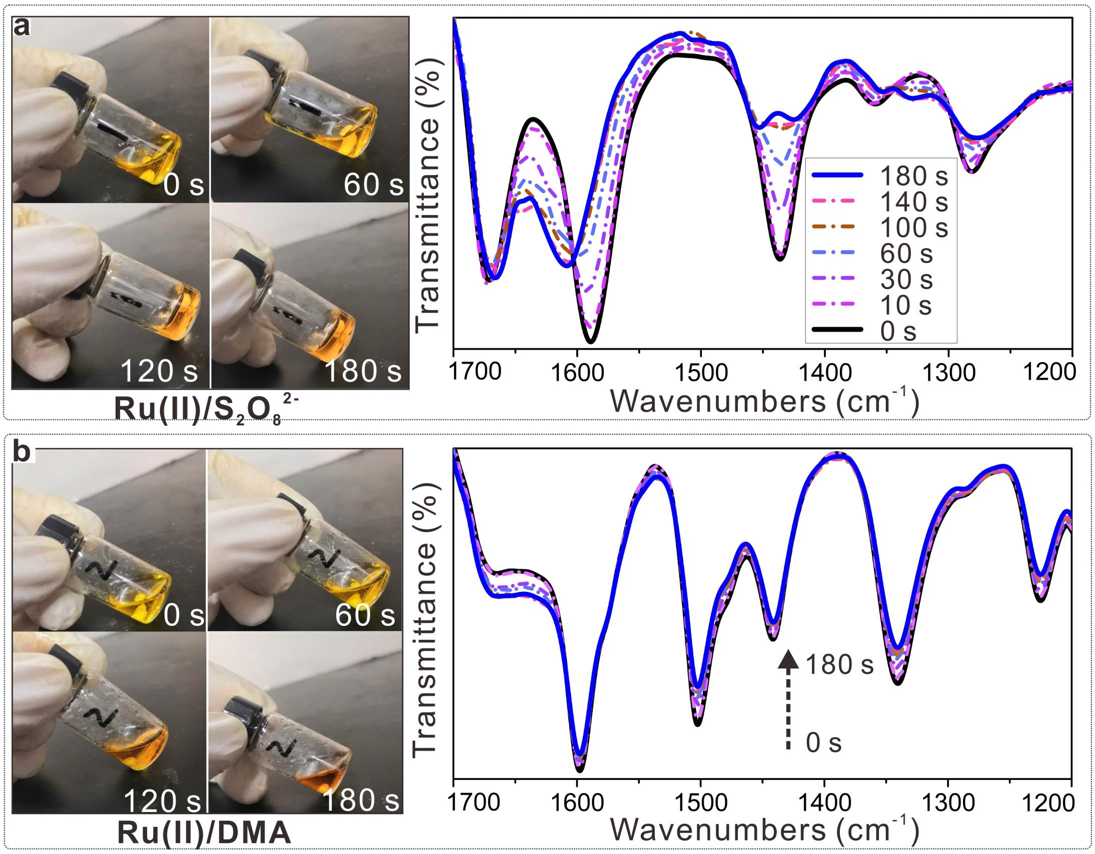

Supplementary Figure 10. Photopolymerization of AAm and MBA at different irradiation time by different catalysts. The catalysts are (**a**) Ru(II)/$S_2O_8^{2-}$ and **(b)** Ru(II)/N, N′-dimethylaniline (DMA), respectively. [AAm] = 2.54 M, [MBA] = 0.7 mM, [Ru(II)] = 31.2 μM and [$S_2O_8^{2-}$] = [DMA] = 131 mM, the irradiation intensity of 15 mW cm^{-2}.

Supplementary Note 6

To further understand the formation mechanism of THVMD hydrogels, another Ru(II)/DMA photoinitiator system was employed to construct the P-N network via the radical polymerization of typical monomers. When exposing the aqueous solution to visible light irradiation, Ru(II) is reduced to Ru(I) by DMA. Meanwhile, DMA is oxidized to the amino radical that can trigger the polymerization reactions. In this study, the experimental conditions were performed at the same conditions, but the initiator systems were different. The reaction processes were monitored by observing the sol-gel transition and in situ FT-IR characterization. As shown in Supplementary Figure 10, we can observe that the hydrogel was formed in 120 s in Ru(II)/$S_2O_8^{2-}$, while the sample was in the liquid state in Ru(II)/DMA with long irradiation. This difference was strongly supported by the FT-IR characterization. The characteristic absorption peak of AAm at ~1440 cm^{-1} rapidly changed within ~100 s light irradiation in Ru(II)/$S_2O_8^{2-}$, but not in Ru(II)/DMA. These achieved results indicated that Ru(II)/$S_2O_8^{2-}$ is a more efficient visible-light-photoinitiator system to trigger the radical polymerization compared with Ru(II)/DMA. In other words, the use of Ru(II)/$S_2O_8^{2-}$ helps fabricate tough hydrogels in seconds.

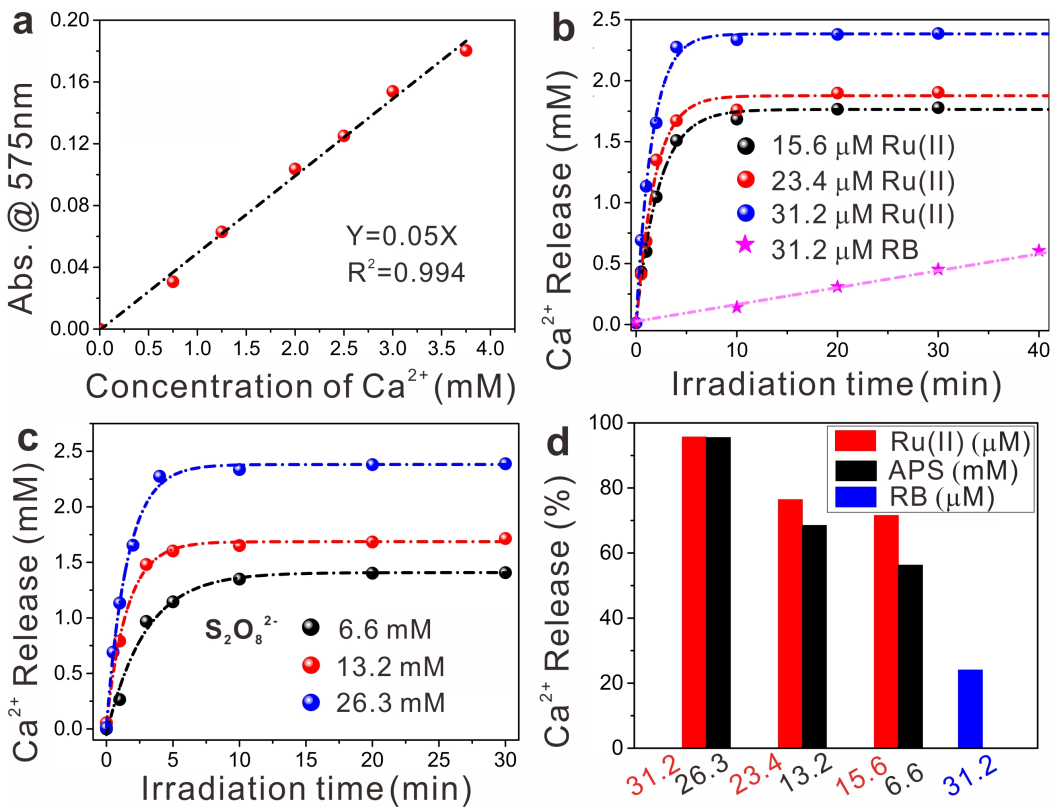

Supplementary Figure 11. Photo-releasing of Ca^{2+} by the catalysis of Ru(II)/$S_2O_8^{2-}$. (**a**) The content of released Ca^{2+} was measured according to the reported literature by Heymann et al[4]. The standard curve of the concentration of free Ca^{2+} vs. UV-vis absorbance at 575 nm. (**b**, **c**) Photoreleasing of Ca^{2+} using riboflavin (RB) and Ru(II)/$S_2O_8^{2-}$ with different concentrations of $S_2O_8^{2-}$. The concentrations of $S_2O_8^{2-}$ in (b) and Ru(II) in (c) were fixed at 26.3 mM and 31.2 μM, respectively. (**d**) The relative release of Ca^{2+} under different conditions in (b, c).

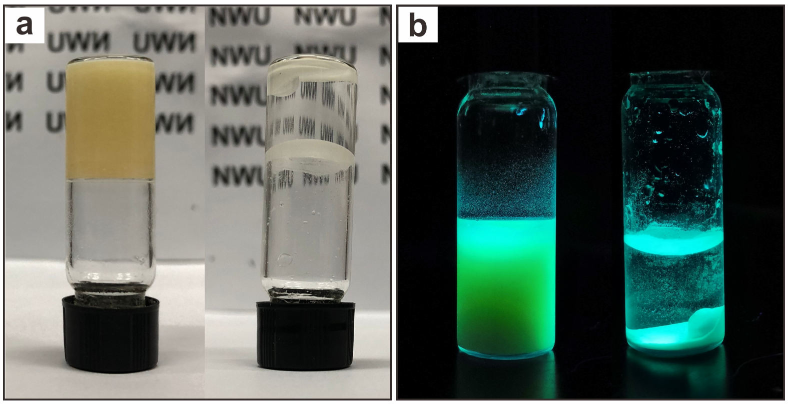

Supplementary Figure 12. Preparing ZnS-contained hydrogel composites (10 wt%). Digital images of the composites made by the visible light-triggered (left) and thermal-induced (right) gelations, respectively, under (a) white and (b) UV light. For the light-triggered reaction, $[S_2O_8^{2-}]$ = 131 mM, [Ru(II)] = 31.2 μM, [AAm] = 2.54 M, [MBA] = 0.7 mM, [ZnS] = 10 wt%, 60 s irradiation at the intensity of 15 mW cm^{-2}. For the thermal-induced reaction, $[S_2O_8^{2-}]$ = 131 mM, [AAm] = 2.54 M, [MBA] = 0.7 mM, [ZnS] = 10 wt%, 3 h heating at 75 ^{o}C.

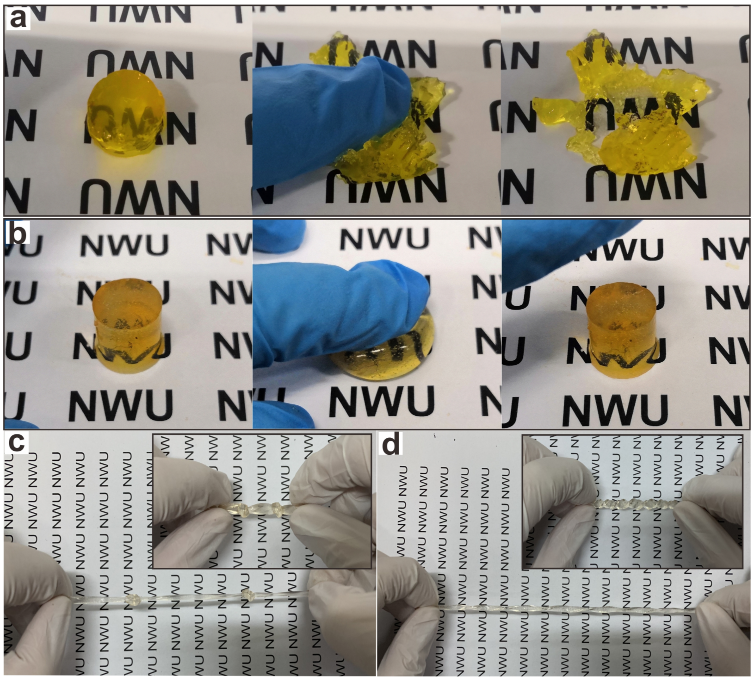

Supplementary Figure 13. Compression and stretchability of hydrogels. (**a**) Ca^{2+}-crosslinked alginate hydrogels and (**b**) THVMD hydrogels before and after compression. (**c**) Knotted and (**d**) twisted THVMD ribbons.

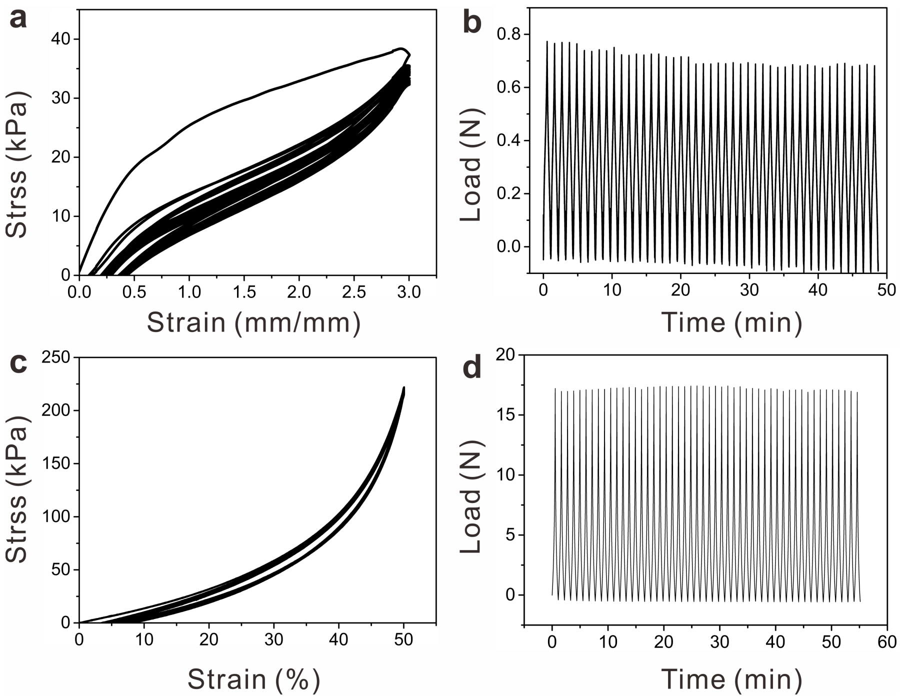

Supplementary Figure 14. Mechanical tests of THVMD hydrogels. (**a**) Tensile stress-strain curves of hydrogels and (**b**) loading force under continuous stretching to a strain of 3. (**c**) Compression stress-strain curves and (**d**) loading force under continuous compressing to a strain of 0.5.

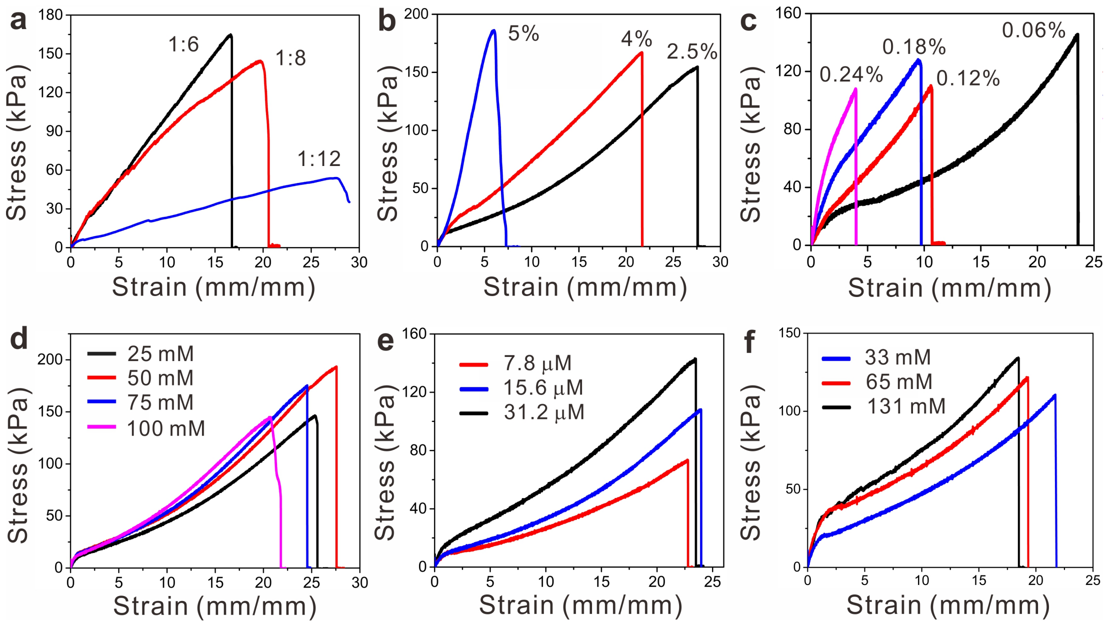

Supplementary Figure 15. Stress-strain curves of different THVMD hydrogels. (**a**) Different weight ratios of mALG to PAAm. (**b**) Different grafting molar ratios of phenol groups on mALG. (**c**) Different molar ratios of MBA to AAm. (**d**-**f**) Different concentrations of EDTA-Ca, Ru(II) and $S_2O_8^{2-}$, respectively. All tensile tests were performed at the stretching speed of 100 mm min^{-1}. Based on these tensile tests, the optimized preparation condition was fixed: [mALG] = 2.25 wt%, grafting ratio of phenol = 2.5 mol.%, [AAm] = 18 wt%, [MBA]: 0.06 wt% of AAm, [EDTA-Ca] = 50 mM, [Ru(II)] = 31.2 μM and [$S_2O_8^{2-}$] = 65 mM. The irradiation intensity was 15 mW cm^{-2}, and the irradiation time was 100 s.

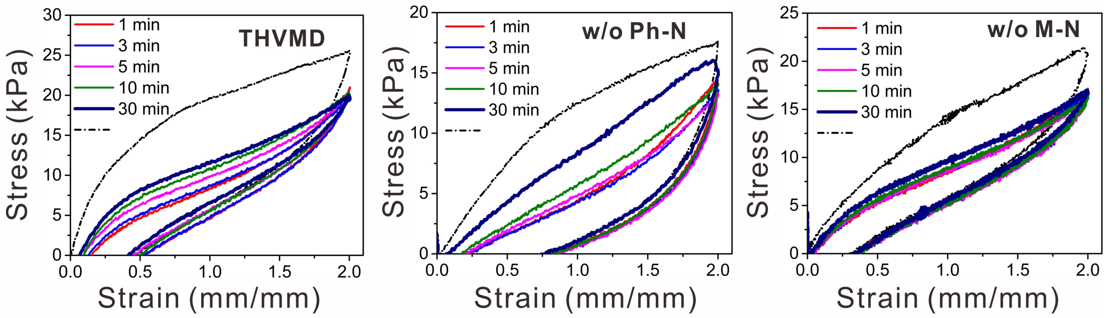

Supplementary Figure 16. Recovery of THVMD hydrogels and the hydrogels without Ph-N and M-N after different waiting times at room temperature. Dash curves show the first cycle of stretching/releasing to the strain of 2.

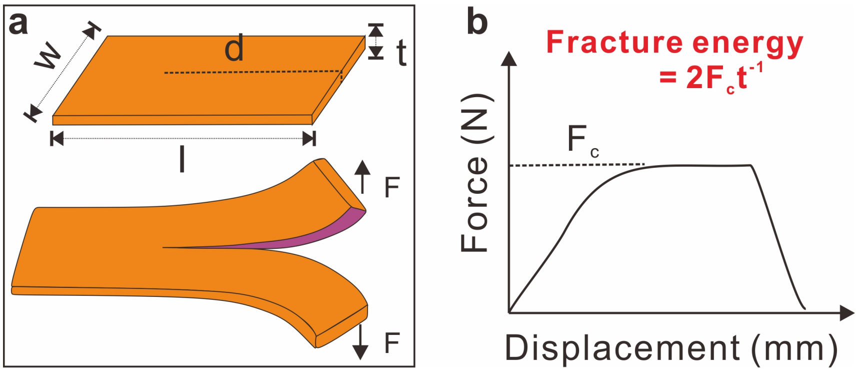

Supplementary Figure 17. The characterization and calculation of fracture energy of THVMD hydrogels. This experiment was conducted according to the literatures[1-3]. (**a**) The geometry of tearing test samples. The length (l), width (w), and thickness (t) of samples are 50 mm, 20 mm, and 2 mm, respectively. The initial notched length is 30 mm. (**b**) The typical force-extension curve of tearing test for THVMD hydrogels. The inset shows the calculation of fracture energy from the obtained maximum tensile force (F_c) and thickness of hydrogels.

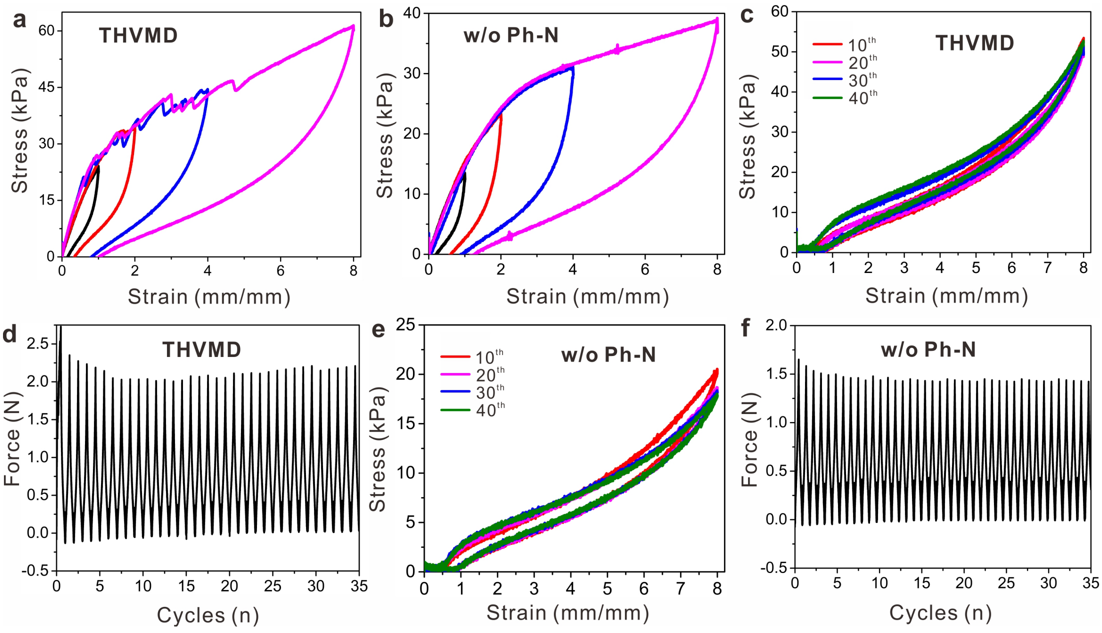

Supplementary Figure 18. Elasticity tests of THVMD hydrogels and the hydrogels without Ph-N. (**a**, **b**) Stress-strain curves with different stretching strains, (**c**-**f**) the repeated cycles of stretching/releasing at the strain of 8 and the corresponding force applied, respectively.

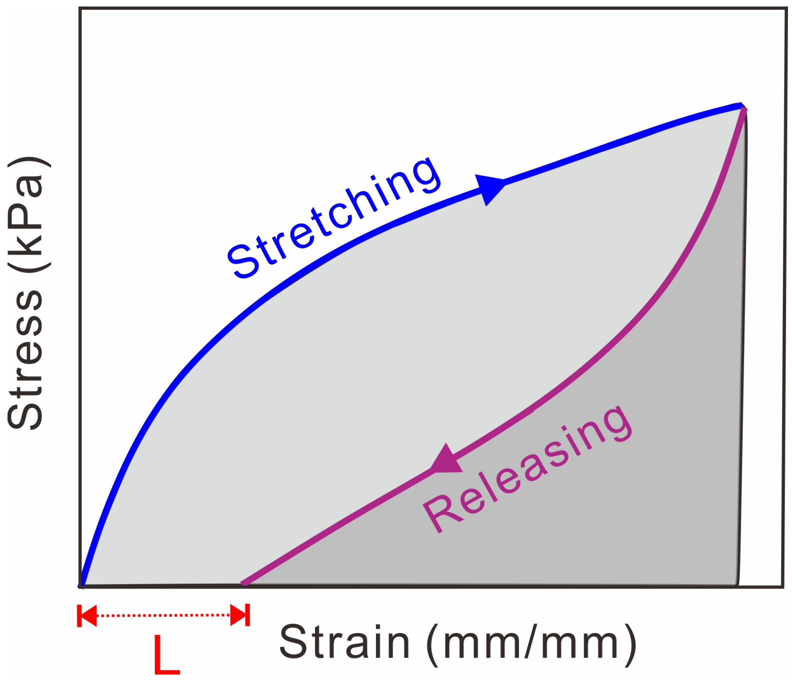

Supplementary Figure 19. Plastic deformation and elasticity properties. Plastic deformation (L) was the strain of hydrogels remained after releasing the stress to 0. Elasticity (%) was defined as the integration ratio of releasing and stretching curves with strain.

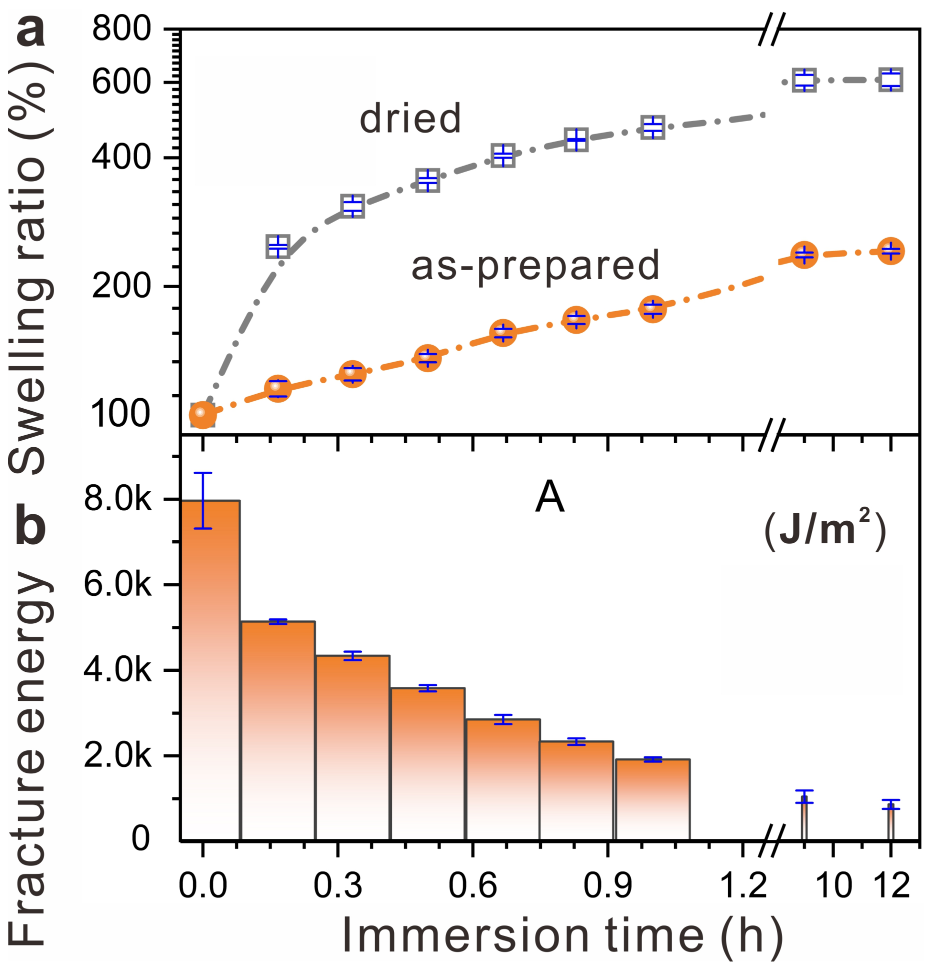

Supplementary Figure 20. The toughness testing of swollen THVMD hydrogels. (**a**) Swelling ratios of dried and as-prepared hydrogels with different immersion times. (**b**) Specific toughness of hydrogels with corresponding immersion times. Before toughness testing, samples were weighed and then immersed into water with pre-determined times at room temperature. After that, the samples were taken out, and excess water was removed by tissue. The swollen samples were weighed again. By comparing the weight between initial and swollen states, swelling ratios were obtained. Figure (a) showed that both dried and as-prepared samples reach nearly equilibrium in 12 h. As for the toughness testing, all swollen samples were tested as same as that of as-prepared samples in Supplementary Figure 17. Figure (b) showed the toughness decreased from ~8.0k to ~1.0k J/m^2 when the swelling ratio increased from 100% to 200%. This result indicated the swelling behavior of hydrogels had a noticeable effect on the toughness of THVMD. Error bars are defined as S.D. (n = 3 independent samples).

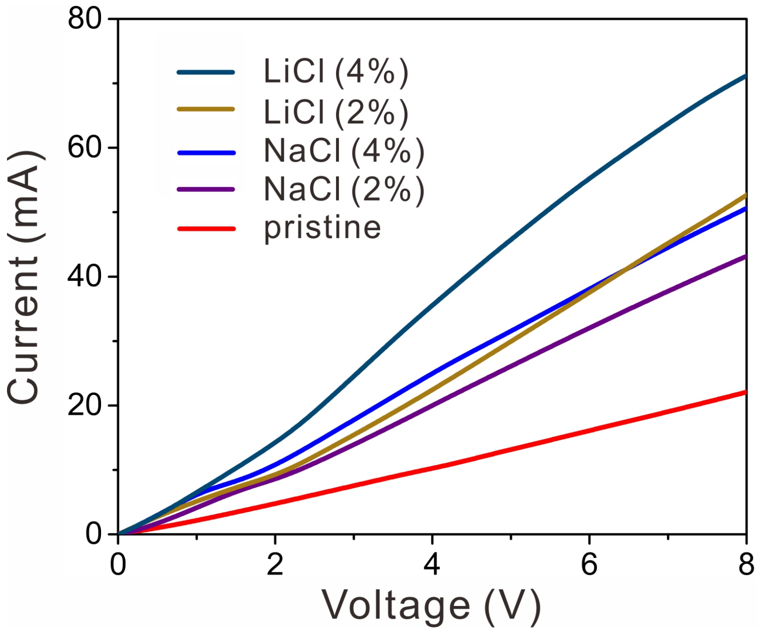

Supplementary Figure 21. Current-Voltage curves of THVMD with different electrolytes added.

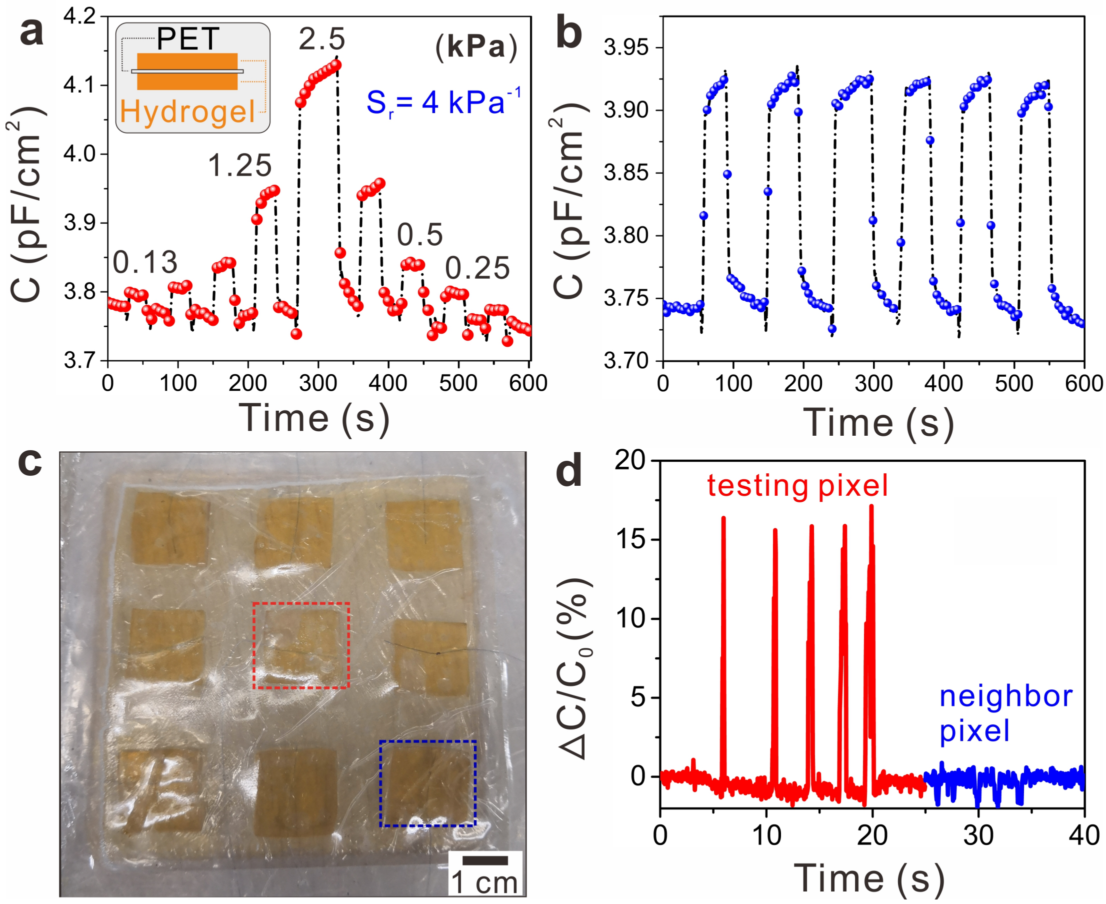

Supplementary Figure 22. Fabrication of capacitive pressure sensors. Capacitances measured at (**a**) different pressures and (**b**) the intermittent pressure of 1.25 kPa, respectively. Inset in (a) shows the structure of the capacitor. (**c**) An integrated three-by-three sensor array. (**d**) Relative Capacitances measured at the labeled testing and neighbor pixels in (c).

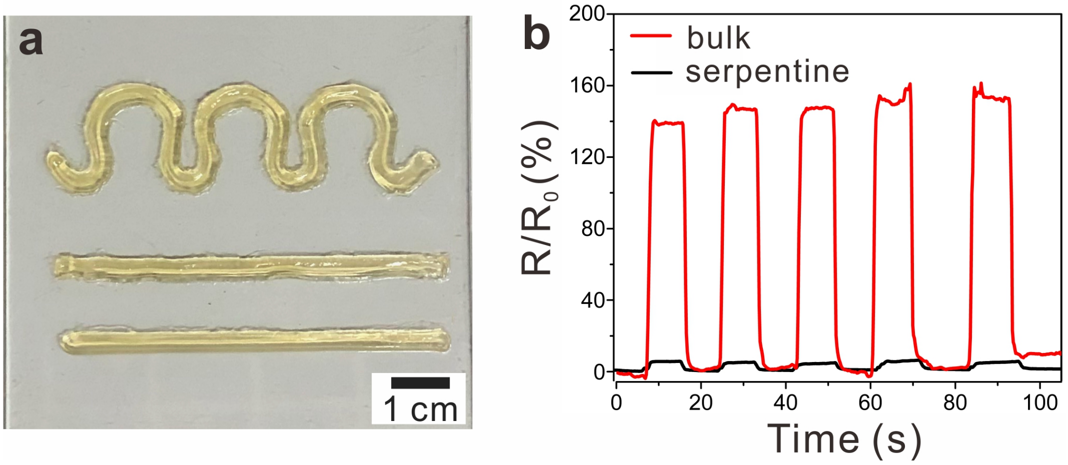

Supplementary Figure 23. Relative resistance of the bulk and serpentine THVMD under certain strains. (**a**) Printed tough hydrogels by the LGDW technique. (**b**) The resistance changes of corresponding hydrogels in Figure (a) with repeated stretching/releasing to a strain of 1.5.

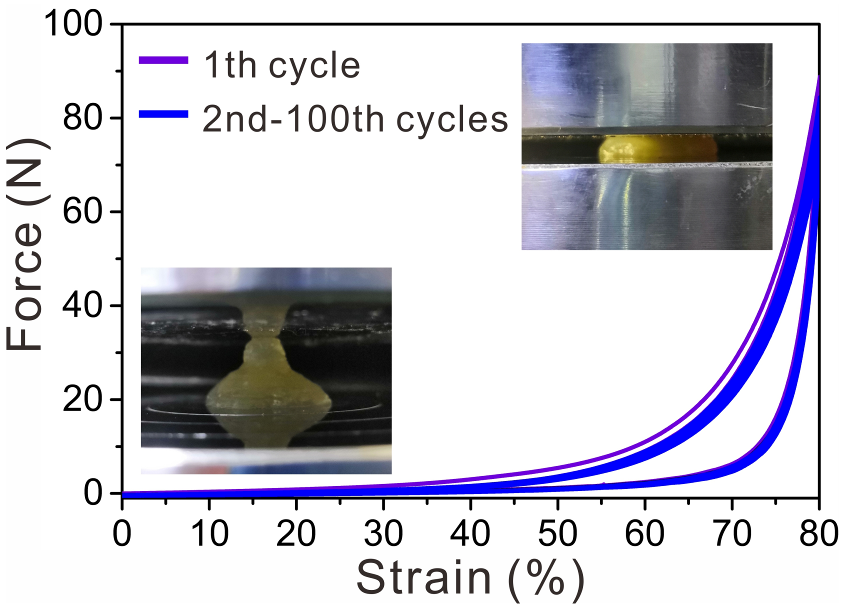

Supplementary Figure 24. The compression test of the 3D printed pyramid-shape hydrogels under continuous 100 cycles of compression-release. The compression tests were carried out at the speed of 10 mm min^{-1}.

Supplementary Table 1. Typically reported strategies for preparing tough hydrogels[1, 5-17]

Components	Method	Time	Strain(Stress)	Printing
ALG/PAAm	UV(50°C)/soaking	>24 h	20 (160 kPa)	NO
ALG/PAAm	UV(50°C)/soaking	>6 h	10~23 (100~1000 kPa)	NO
Agar/PAAm	Heating(90°C)/UV	1~2 h	22 (180 kPa)	~cm
CS/PAAm	UV(60°C)/soaking	24h	4(6 MPa)	NO
PVA/PAAm	Heating(60°C)/UV	>2 h	5 (200 kPa)	NO
PAA/PAAm	Fe(II)-Mediated	5~10 min	N/A	~μm
ALG/PAAm/GO	Heating(80°C)/soaking	>240 h	10 (150 kPa)	NO
Polyion complex	UV/dialysis	>24 h	7 (3.5 MPa)	NO
SPAAC/PC	UV	12 min	N/A	NO
ALG/PAAm/SBA-15	UV/soaking	>24 h	28 (140 kPa)	NO
PAMPS/PAAm	UV/soaking	>24 h	0.92 (17.2 MPa)	NO
Agar/PAAAAc-Fe^{3+}	Heating(90°C)/UV	>72 h	14 (1.6 MPa)	NO
ALG/PEG	UV	>10 min	4.5 (259 kPa)	~μm
OA/GelMA	UV	>35 min	0.5 (100 kPa)	NO
**This work**	**Visible light, RT**	**45 s**	**28 (200 kPa)**	**~μm**

Supplementary References

1. Sun, J.-Y., Zhao, X., *et al.* Highly stretchable and tough hydrogels. *Nature* **489,** 133 (2012).

2. Sun, T. L., Kurokawa, T., *et al.* Physical hydrogels composed of polyampholytes demonstrate high toughness and viscoelasticity. *Nat. Mater.* **12,** 932 (2013).

3. Bai, R., Yang, J. & Suo, Z. Fatigue of hydrogels. *Eur. J. Mech. A-Solid* **74,** 337-370 (2019).

4. Heymann, R. R., Thum, M. D., *et al.* Visible light initiated release of calcium ions through photochemical electron transfer reactions. *Photoch. Photobio. Sci.* **16,** 1003-1008 (2017).

5. Yang, C. H., Wang, M. X., *et al.* Strengthening Alginate/Polyacrylamide Hydrogels Using Various Multivalent Cations. *ACS Appl. Mater. Inter.* **5,** 10418-10422 (2013).

6. Choi, S., Choi, Y. j., *et al.* Supertough hybrid hydrogels consisting of a polymer double-network and mesoporous silica microrods for mechanically stimulated on-demand drug delivery. *Adv. Funct. Mater.* **27,** 1703826 (2017).

7. Ge, G., Zhang, Y., *et al.* Stretchable, transparent, and self-patterned hydrogel-based pressure sensor for human motions detection. *Adv. Funct. Mater.* **28,** 1802576 (2018).

8. Chen, Q., Zhu, L., *et al.* A robust, one-pot synthesis of highly mechanical and recoverable double network hydrogels using thermoreversible sol-gel polysaccharide. *Adv. Mater.* **25,** 4171-4176 (2013).

9. Gong, J. P., Katsuyama, Y., *et al.* Double-network hydrogels with extremely high mechanical strength. *Adv. Mater.* **15,** 1155-1158 (2003).

10. Hong, S., Sycks, D., *et al.* 3D printing of highly stretchable and tough hydrogels into complex, cellularized structures. *Adv. Mater.* **27,** 4035-4040 (2015).

11. Luo, F., Sun, T. L., *et al.* Oppositely charged polyelectrolytes form tough, self-healing, and rebuildable hydrogels. *Adv. Mater.* **27,** 2722-2727 (2015).

12. Ma, S., Yan, C., *et al.* Continuous surface polymerization via Fe(II)-mediated redox reaction for thick hydrogel coatings on versatile substrates. *Adv. Mater.* **30,** 1803371 (2018).

13. Yang, Y., Wang, X., *et al.* Highly elastic and ultratough hybrid ionic–covalent hydrogels with tunable dtructures and mechanics. *Adv. Mater.* **30,** 1707071 (2018).

14. Chen, Q., Yan, X., *et al.* Improvement of mechanical strength and fatigue resistance of double network hydrogels by ionic coordination interactions. *Chem. Mater.* **28,** 5710-5720 (2016).

15. Jeon, O., Shin, J.-Y., *et al.* Highly elastic and tough interpenetrating polymer network-structured hybrid hydrogels for cyclic mechanical loading-enhanced tissue engineering. *Chem. Mater.* **29,** 8425-8432 (2017).

16. Brown, T. E., Silver, J. S., *et al.* Secondary photocrosslinking of click hydrogels to probe myoblast mechanotransduction in three dimensions. *J. Am. Chem. Soc.* **140,** 11585-11588 (2018).

17. Fan, J., Shi, Z., *et al.* Mechanically strong graphene oxide/sodium alginate/polyacrylamide nanocomposite hydrogel with improved dye adsorption capacity. *J. Mater. Chem. A* **1,** 7433-7443 (2013).
