# Supplementary material for: Visible-light-assisted multimechanism design for one-step engineering tough hydrogels in seconds
Source: Nat Commun. 2020 Oct 5;11:4694. doi: 10.1038/s41467-020-18145-w (PMC7536405; doi:10.1038/s41467-020-18145-w)
Supplement: Supplementary file 2 — Description of Additional Supplementary Files [file 41467_2020_18145_MOESM2_ESM.pdf]

**Description of Additional Supplementary Files**

File Name: Supplementary Movie 1
Description: 3D extrusion printing of tough pyramid hydrogel with the THVMD strategy

File Name: Supplementary Movie 2
Description: Operating the integrated device by a tough hydrogel-based touching sensor

File Name: Supplementary Movie 3
Description: Stretching, bending, and twisting the as-prepared flexible electroluminescent unit
